# Selective and brain-penetrant ACSS2 inhibitors target breast cancer brain metastatic cells

**DOI:** 10.3389/fphar.2024.1394685

**Published:** 2024-05-16

**Authors:** Emily M. Esquea, Lorela Ciraku, Riley G. Young, Jessica Merzy, Alexandra N. Talarico, Nusaiba N. Ahmed, Mangalam Karuppiah, Anna Ramesh, Adam Chatoff, Claudia V. Crispim, Adel A. Rashad, Simon Cocklin, Nathaniel W. Snyder, Joris Beld, Nicole L. Simone, Mauricio J. Reginato, Alexej Dick

**Affiliations:** ^1^ Department of Biochemistry and Molecular Biology, Drexel University College of Medicine, Philadelphia, PA, United States; ^2^ Department of Cardiovascular Sciences, Temple University Lewis Katz School of Medicine, Philadelphia, PA, United States; ^3^ Department of Microbiology and Immunology, Drexel University College of Medicine, Philadelphia, PA, United States; ^4^ Department of Radiation Oncology, Sidney Kimmel Cancer Center, Thomas Jefferson University, Philadelphia, PA, United States; ^5^ Cancer Risk and Control Program, Philadelphia, PA, United States; ^6^ Translational Cellular Oncology Program, Sidney Kimmel Cancer Center, Thomas Jefferson University, Philadelphia, PA, United States

**Keywords:** cancer, metabolism, breast cancer, brain metastasis, acetate, acetyl-CoA, ACSS2, computational-aided drug design font: italic formatted: left

## Abstract

Breast cancer brain metastasis (BCBM) typically results in an end-stage diagnosis and is hindered by a lack of brain-penetrant drugs. Tumors in the brain rely on the conversion of acetate to acetyl-CoA by the enzyme acetyl-CoA synthetase 2 (ACSS2), a key regulator of fatty acid synthesis and protein acetylation. Here, we used a computational pipeline to identify novel brain-penetrant ACSS2 inhibitors combining pharmacophore-based shape screen methodology with absorption, distribution, metabolism, and excretion (ADME) property predictions. We identified compounds AD-5584 and AD-8007 that were validated for specific binding affinity to ACSS2. Treatment of BCBM cells with AD-5584 and AD-8007 leads to a significant reduction in colony formation, lipid storage, acetyl-CoA levels and cell survival *in vitro*. In an *ex vivo* brain-tumor slice model, treatment with AD-8007 and AD-5584 reduced pre-formed tumors and synergized with irradiation in blocking BCBM tumor growth. Treatment with AD-8007 reduced tumor burden and extended survival *in vivo*. This study identifies selective brain-penetrant ACSS2 inhibitors with efficacy towards breast cancer brain metastasis.

## Introduction

Breast cancer is the most commonly diagnosed cancer in women worldwide (Arnold et al., 2022), with an estimated 10%–35% of breast cancer patients developing metastasis to the brain (Tsukada et al., 1983; Patanaphan et al., 1988; Willett et al., 2015). Breast-cancer brain metastases (BCBM) currently represent an incurable event (Wang et al., 2021), with over 80% of patients succumbing to end-stage disease within a year of diagnosis (Dawood et al., 2012; Arnold et al., 2022). Limited therapeutic interventions, including surgical resection, whole brain radiation, and chemotherapy, are often ineffective and yield detrimental effects on healthy brain tissue, thereby profoundly diminishing the quality of life of affected patients (Leone and Leone, 2015; Wang et al., 2021; Watase et al., 2021). Thus, there is an urgent need to identify small molecules that can block tumor growth in the brain and extend survival of patients with brain metastasis.

Tumor cells that grow in the brain are situated within a nutrient-depleted and hypoxic tumor brain microenvironment; thus, these cancer cells must adapt metabolically to survive (Badr et al., 2020; Aili et al., 2022). These growing tumor cells undergo processes of metabolic reprogramming that allows for the survival, growth, and progression of these tumorsin the brain microenvironment. However, these metabolic adaptations can represent vulnerabilities that may be exploited therapeutically (Badr et al., 2020; Aili et al., 2022). The acetate dependency of brain growing tumor cells is a unique characteristic of these tumors that represents a promising metabolic-related therapeutic target (Yoshii et al., 2009a; Yoshii et al., 2009b). Acetate is an alternative carbon source to generate nuclear-cytoplasmic acetyl-CoA via the nuclear-cytosolic enzyme acetyl-CoA synthetase 2 (ACSS2) (Yoshii et al., 2009a; Yoshii et al., 2009b; Ling et al., 2022). ACSS2 derived acetyl-CoA plays a critical role in cellular energetics and anabolism as precursor for *de novo* lipid synthesis and is an acyl-donating substrate for protein and histone acetylation (Feron, 2019; Ling et al., 2022). ACSS2 has been shown to play a critical role in tumor cell growth in various cancers, including breast (Comerford et al., 2014; Schug et al., 2015; Miller et al., 2021a) and brain cancers (Ciraku et al., 2022). In particular, ACSS2 may serve as an attractive therapeutic target for tumors in the brain, such as glioblastoma and brain metastasic tumors, due to the preferential use of acetate in these tumors (Mashimo et al., 2014). Importantly, genetically targeting ACSS2 in brain tumors has previously been shown to block tumorigenesis (Li et al., 2017; Ciraku et al., 2022). Additionally, ACSS2 null mice are phenotypically normal, without embryonical or developmental deficiencies, suggesting that ACSS2 may be a non-essential gene under normal conditions (Huang et al., 2018), making ACSS2 an attractive cancer-specific target. Several small molecules targeting ACSS2 have been identified and tested in liver (Comerford et al., 2014) and breast cancer models (Schug et al., 2015; Miller et al., 2021a; Miller et al., 2021b). Currently, MTB-9655, the first oral ACSS2 inhibitor, is in phase I clinical trials for advanced solid tumors (Perets et al., 2022). However, to our knowledge, there are currently no small molecule ACSS2 inhibitors that can cross the blood-brain barrier (BBB) for treating cancers in the brain.

In this study, we sought to discover novel, pharmacologically stable, small-molecule inhibitors of ACSS2 that are able to cross the BBB. Utilizing a previously validated computational workflow (Tuyishime et al., 2014; Karadsheh et al., 2020; Xu et al., 2022; Zhang et al., 2022; Zhang et al., 2023) with additional brain and CNS-specific parameters, we identified several new chemotypes in the ACSS2 inhibitor class. These analog ACSS2 inhibitors exhibit drug-like properties, coinciding with computational predictions. We show that these inhibitors can suppress BCBM tumor cell growth *in vitro* and *ex vivo* and cross the BBB and block BCBM growth *in vivo*. These first-in-class BBB-permeable ACSS2 inhibitor analogs provide scaffolds for further optimization of ACSS2-targeting chemotypes and enable improved cancer treatments in the brain.

## Results

### A computational pipeline for predicting drug-like properties for the discovery of brain-permeable ACSS2 inhibitors

**FIGURE 1:**
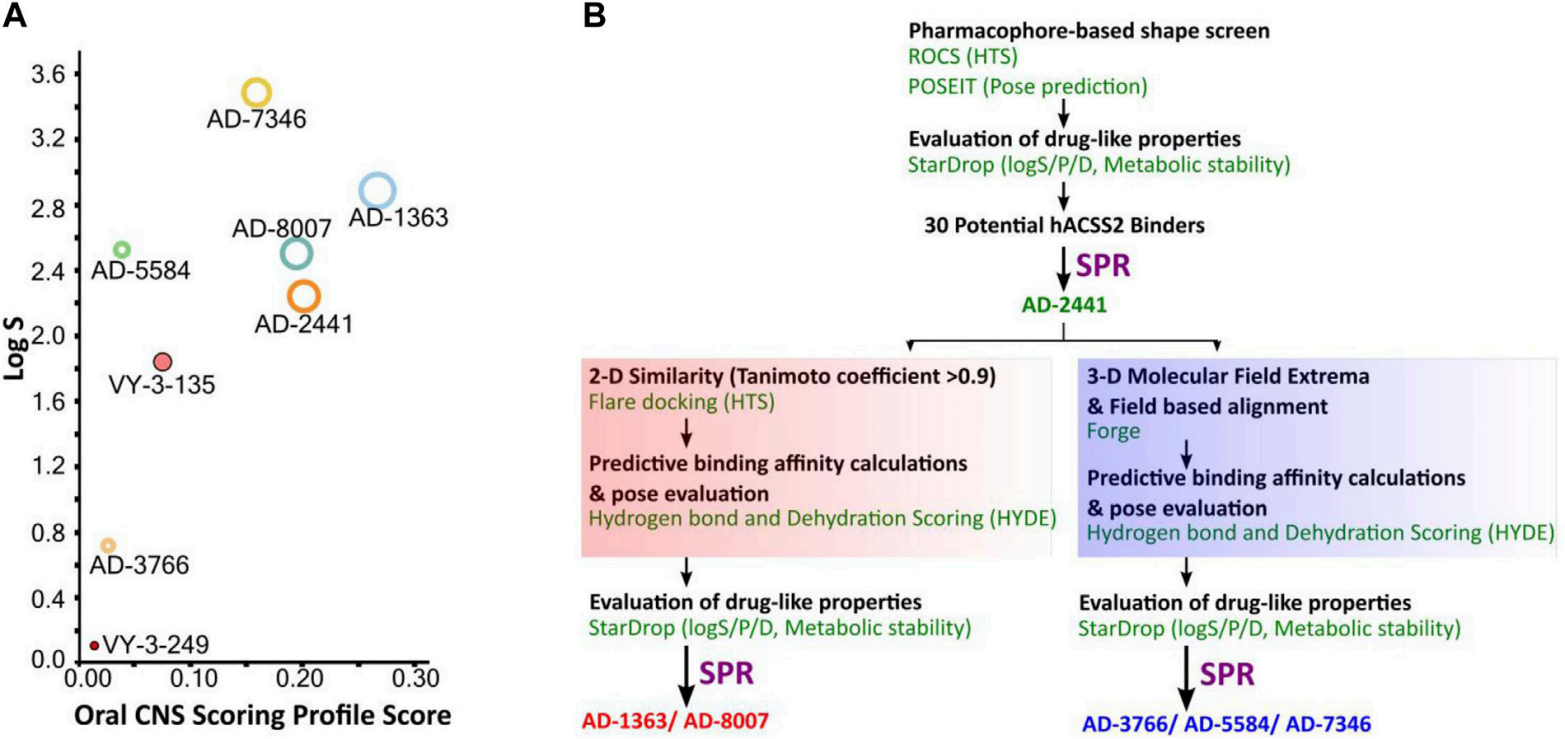
Computational pipeline and predicted drug-like properties for the discovery of AD-2441 and its analogs. **(A)** Oral CNS Scoring profile score vs logS of AD-2441 and its analogs, including two currently known control inhibitors, VY-3-249 and VY-3-135. Plot showing the StarDrop V7 (Optibrium, Ltd., Cambridge, United Kingdom)–derived logS *versus* a multimetric oral CNS profile score. Score composition and importance of each contributor: logS = 0.8, logP = 0.6, logD = 0.6, BBB category = 0.55, BBB log(brain:blood) = 0.55, P-gp category = 0.5, HIA = 0.4, hERG pIC50 = 0.2, 2D6 affinity category = 0.16, 2C9 pKi = 0.16, PPB90 category = 0.1. The size of the circle correlates with the corresponding score or probability. **(B)** Computational and SPR-based validation pipeline for the discovery of novel ACSS2 inhibitors.

The quinoxaline-based chemotype VY-3-249 targeting ACSS2 previously identified (Comerford et al., 2014) is not predicted to traverse the BBB as it has low oral CNS scoring profiles (Figure 1A, Supplementary Figure S1). A new derivative of VY-3-249, compound VY-3-135, a quinoxaline-based chemotype was recently shown to have increased potency and stability compared to VY-3-249 (Miller et al., 2021a), yet it is not predicted to cross the BBB (Figure 1A, Supplementary Figure S1). Thus, we sought to identify novel ACSS2 inhibitor chemotypes with BBB permeability in order to target brain tumors. We utilized a pharmacophore-based shape screen methodology (Segall et al., 2009; Segall, 2012; Hunt et al., 2018), further processed through validation of binding poses and computational prediction of (Segall et al., 2009) absorption, distribution, metabolism, and excretion (ADME) properties and other drug-like features (Figure 1B). These *in silico* predictions were conducted employing StarDrop V7, with the incorporation of the oral central nervous system (CNS) drug profile and an auxiliary parameter for logD (Tuyishime et al., 2014; Tuyishime et al., 2016; Tyzack et al., 2016). The oral CNS drug profile comprises numerous models integrated into an overall score by a probabilistic scoring algorithm. This scoring system spans from 0 to 1, where a score of 0 suggests a non-drug-like compound, while 1 indicates the paradigm of a drug. This computational pipeline and stringent drug-like properties filtering distilled our initial molecule pool to 30 potential ACSS2 binders.

### Validating the binding affinity and predicted metabolic stability of ACSS2 inhibitors

**FIGURE 2:**
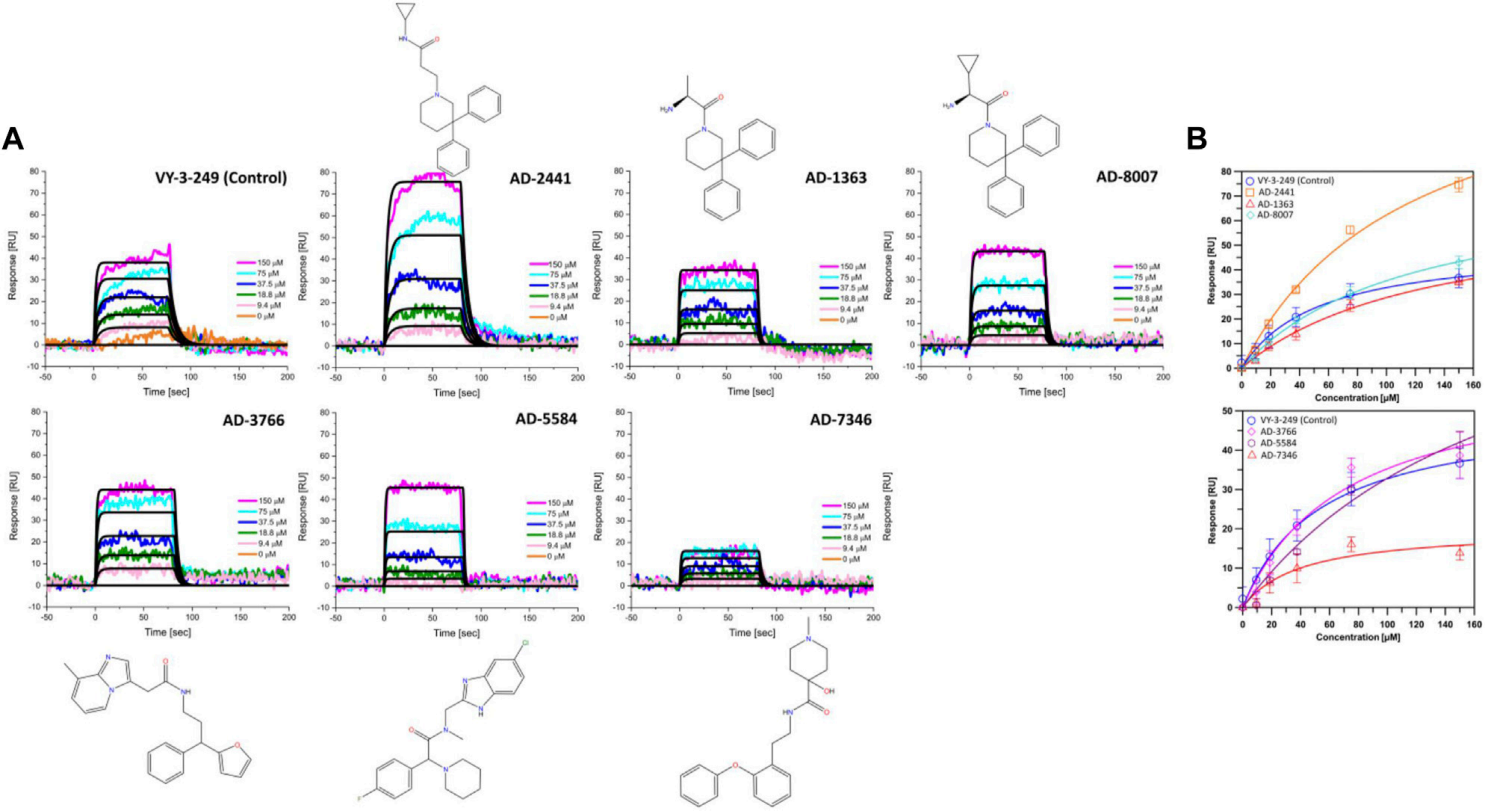
Representative sensorgrams and structures for AD-2441 and its analogs binding to ACSS2. **(A)** Sensorgrams of ACSS2 binders. Colored lines represent collected data from the dilution series, whereas black lines represent a fit to a 1:1 binding model—interaction parameters derived from a triplicate (n = 3) of data given in Supplementary Table S1. **(B)** Binding isotherms of AD-2441 and analogs. Binding isotherms are derived from panel **(A)**. Experiments were performed in triplicate, and data displayed with standard deviations (SD with n = 3).

**FIGURE 3:**
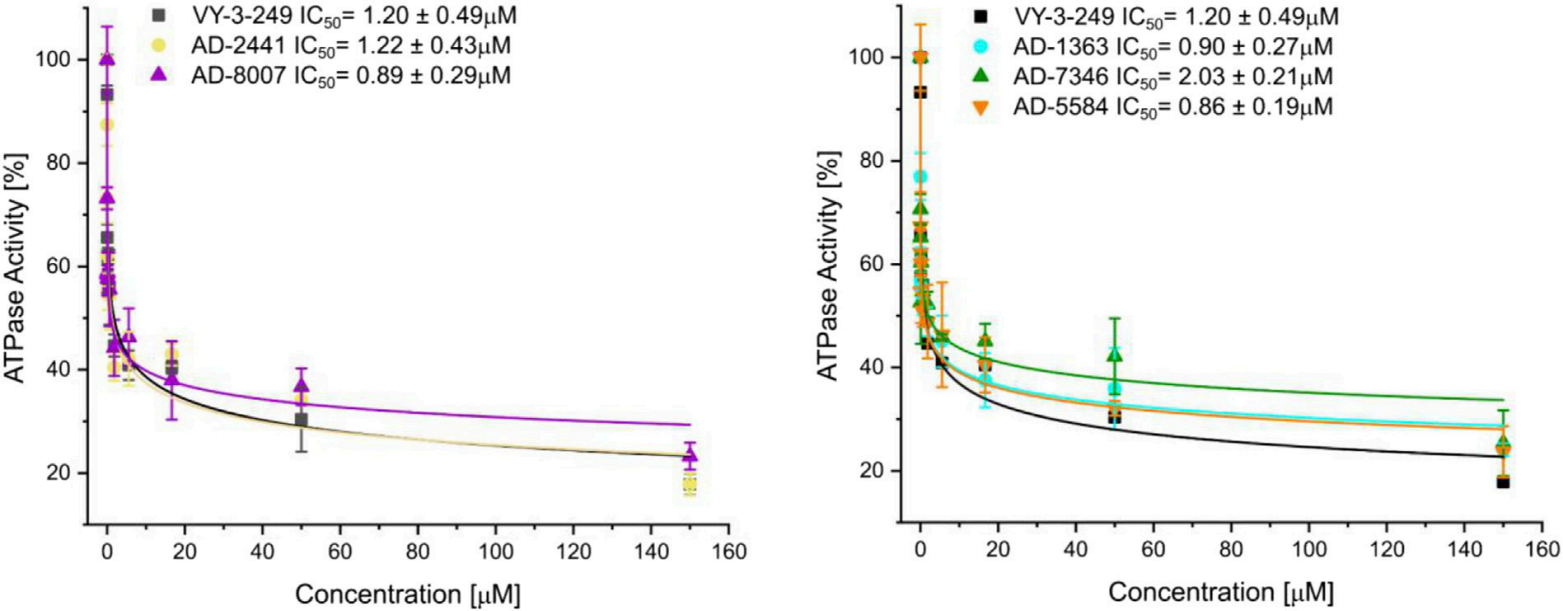
Fluorescent polarisation-based assay for measurement of inhibition of ATP to AMP conversion of ACSS2 by selected ACSS2 inhibitors. Experiments were performed using the Tecan Spark multimode microplate reader with 100 nM ACSS2 and varying compound concentrations. Experiments were performed in triplicate, and data displayed with standard deviations (SD with n = 3).

From the 30 potential ACSS2 inhibitors from our computational workflow (Figure 1B), we next tested whether these compounds bind to their intended target, human ACSS2, in order to inhibit its function. To test the binding affinity of the potential ACSS2 inhibitors, we used surface plasmon resonance (SPR) interaction analysis for directing binding affinity and kinetic and a fluorescent-based adenosine triphosphatase (ATPase) inhibition assay to ascertain *in vitro* ATPase inhibition (IC_50_). The outcome identified six candidates exhibiting low-micromolar affinities and IC_50s_ in the high nanomolar range (Figure 2; Figure 3, and Supplementary Table S1).

**FIGURE 4:**
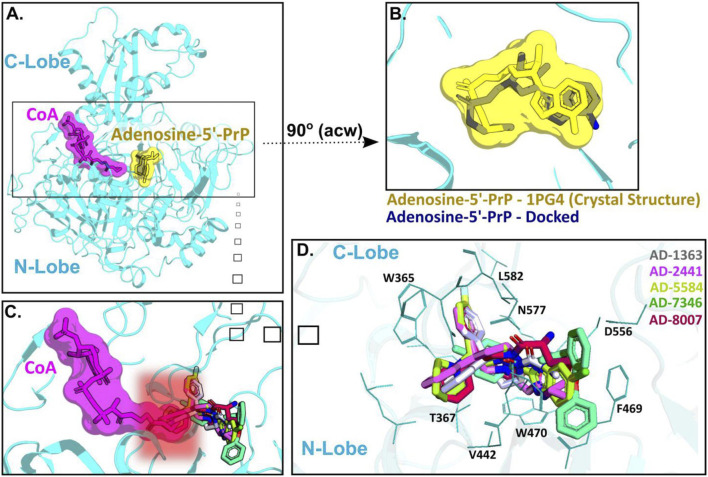
AD-2441 and its analogs are predicted to bind ACSS2 within the nucleotide-binding pocket and stabilized by various hydrophobic and polar contacts and aromatic stacking interactions. Docking calculations were performed into a homology model (using swissmodel.expasy.org) (Waterhouse et al., 2018; Studer et al., 2020) of ACSS2 based on the crystal structure of *Salmonella enterica* acetyl-CoA synthetase in complex with cAMP and Coenzyme A (PDB: 5JRH). **(A)** Homology model of ACSS2 with superimposed CoA (purple) and Adenosine-5′-propylphosphate (yellow, extracted from PDB: 1PG4). **(B)** Superimposed crystal structure of Adenosine-5′-propylphosphate (yellow, extracted from PDB: 1PG4) and docked (DiffDock and Flare version 5 minimized) Adenosine-5′-propylphosphate pose (blue). **(C)** All docked compounds are predicted to bind within the Adenosine-5′-propylphosphate site and additionally sterically interfere with CoA (purple) binding indicated by the red helo. **(D)** Close-up view of binding poses of AD-1363, AD-2441, AD-5584, AD-7346, and AD-8007 between the C- and N-Lobe of ACSS2.

ACSS2 belongs to an enzyme family known for initiating reactions that generate adenosine monophosphate (AMP) through a two-phase process (Gulick et al., 2003). Initially, acyl-AMP is formed while simultaneously releasing pyrophosphate and the intermediate stage involves the formation of acetyl-AMP. Subsequently, Coenzyme A (CoA) replaces AMP, producing the endproduct acetyl-CoA (Gulick et al., 2003). ACSS2 comprises a C-terminal and N-terminal lobe with CoA and acetyl-AMP binding between those two domains (Figure 4A). For our docking approach, we first compared the crystal structure of Adenosine-5′-propylphosphate (from PDB: 1PG4) with our docked Adenosine-5′-propylphosphate pose, to validate the quality of the docking approach (Figure 4B) and highlight the accuracy of our homology model of ACSS2 for compound docking.

Subsequent docking calculations predict that all six analogs bind near the acetyl-AMP site (Figure 4C), potentially mimicking a short-lived transition state vital for ACSS2 function (Jezewski et al., 2021). In addition, the binding of our compounds to ACSS2 most likely interferes sterically with CoA binding, therefore the acetyl transfer (Figure 4C, highlighted by the red helo). Besides hydrophobic (Val442, Leu582) and polar interactions (Asn577, Asp556), such as hydrogen bonds and salt bridges, a notable key feature of all compounds were π-π interactions via their bifurcated aromatic moieties with Trp365, Phe469, and Trp470 (Figure 4D). Importantly, these potential inhibitors showcased predicted improved drug-like characteristics compared to those of control compounds with most notable metabolic stability (Supplementary Figure S2A–C) and BBB permeability (Figure 1A, Supplementary Figure S1C).

Orally administered drugs undergo first-pass effect, a common occurrence in which drugs become metabolized, typically in the liver, and the concentration of the active drug is reduced (Pond and Tozer, 1984). Due to the first-pass effect, the metabolic stability of compounds can limit the concentration of these compounds in the bloodstream, directly effecting drug efficacy. Thus, we sought to computationally investigate if our identified ACSS2 inhibitors are predicted to have increased metabolic stability compared to previously established ACSS2 inhibitors, VY-3-249 and VY-3-135. We utilized a computational analysis, applying the P450 module in StarDrop V7 software to predict each compound’s primary metabolizing Cytochrome P450 isoforms using the WhichP450™ model (Tyzack et al., 2016; Hunt et al., 2018; Karadsheh et al., 2020). This was followed by an estimation of the compound’s affinity to that isoform by applying the HYDE function in SeeSAR (Reulecke et al., 2008) (Supplementary Figure S2). This methodology has been used successfully in predicting and improving the metabolic stability of HIV-1 inhibitory compounds (Tuyishime et al., 2014; Karadsheh et al., 2020; Xu et al., 2022; Zhang et al., 2022; Zhang et al., 2023). The CYP3A4/2D6 isoforms act as the major metabolizing enzyme for all compounds, including the control (Supplementary Figure S2A). We investigated the predicted metabolic lability of our compounds with the CY3A4 isoform, gauging the overall composite site lability (CSL) score and the number of labile sites. The CSL score amalgamates the labilities of individual sites within the compound, providing insight into the efficiency of the molecule’s metabolism (Tyzack et al., 2016; Hunt et al., 2018; Karadsheh et al., 2020). Compared to the control VY-3-249, our compounds displayed lower labile sites and CSL scores, suggesting increased metabolic stability (Supplementary Figure S2B,C). While the CSL score and number of labile sites provide useful information, they assume all compounds bind with similar affinity to the CYP3A4 isoform. However, other factors influence metabolic stability, such as the binding affinity to the CYP3A4 isoform, compound reduction rate, and inherent compound properties like size and lipophilicity (Tyzack et al., 2016; Hunt et al., 2018; Karadsheh et al., 2020).

Consequently, we performed predictive binding affinity calculations using the hydrogen bond and dehydration (HYDE) energy scoring function in SeeSAR 12.1 (Reulecke et al., 2008) with the structure of the human CYPA4 bound to an inhibitor (PDB: 4D78) (Samuels and Sevrioukova, 2020). The HYDE scoring function in SeeSAR offers a range of affinities, stipulating an upper and lower limit. By integrating the CSL scores, labile sites, and predicted CYP3A4/2D6 affinity, our analysis suggests that compounds AD-1363, AD-2441, AD-5584, and AD-8007 may have improved metabolic stability compared to control compounds (Supplementary Figure S2B,C). Additionally, we confirmed via SPR that two of our lead compounds AD-5584 and AD-8007 specifically bind ACSS2 and not the mitochondrial isoform hACSS1 (Supplementary Figure S2D).

### Evaluation of ACSS2 inhibitors on tumor cell growth and lipid content *in-vitro*

**FIGURE 5:**
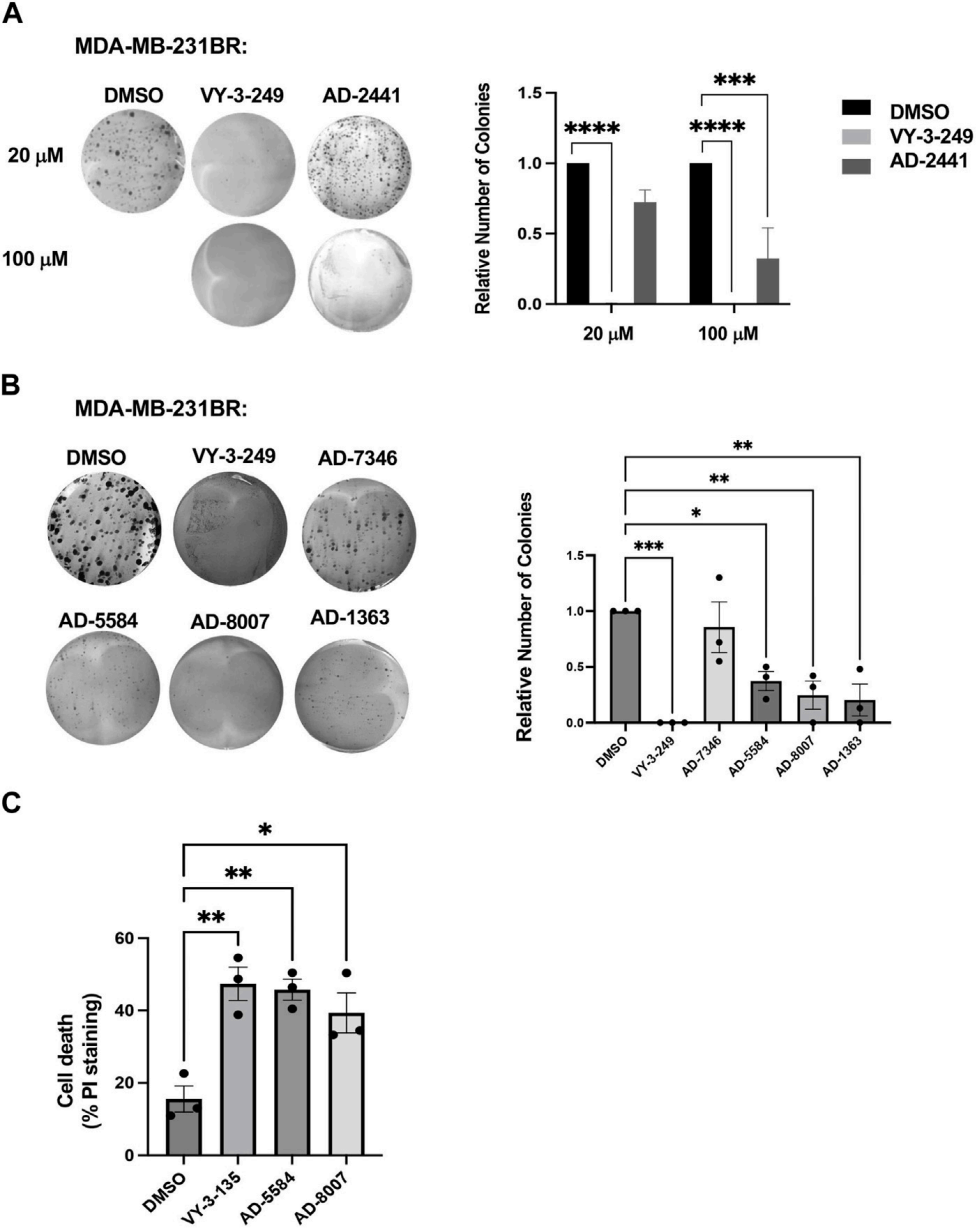
*In Vitro* effect of ACSS2 inhibitors on MDA-MB-231BR cells cell survival. **(A)** Representative images of MDA-MB-231BR cells treated with ACSS2 inhibitors for 48 h at 100 μM and seeded in clonogenic cells survival assay stained with crystal violet. Average colony formation quantified and presented as average from three independent experiments. Two-way ANOVA reported as mean ± SEM. ***p < 0.001, ****p < 0.0001. **(B)** Representative images of MDA-MB-231BR cells treated with ACSS2 inhibitors for 48 h and seeded clonogenic cells survival assay stained with crystal violet. Average colony formation quantified and presented as average from three independent experiments. One-way ANOVA reported as mean ± SEM. *p < 0.05, **p < 0.01, ***p < 0.001. **(C)** Quantification of cell death as measured by Propidum Idodine (PI)+ cells detected by flow cytometry analysis of MDA-MB-231BR cells treated with ACSS2 inhibitors at 100 μM for 48 h. One-way ANOVA reported as mean ± SEM. *p < 0.05, **p < 0.01, ***p < 0.001.

Having identified compounds that bind to and inhibit ACSS2 *in vitro*, we sought to determine whether they had biological effects on brain-tropic breast cancer cells. For this study, we utilized brain-trophic breast cancer cells, MDA-MB-231BR or 4T1BR, which are derived from parental breast cancer cells MDA-MB-231 or 4T1, respectively. The BR or brain trophic derivatives have been selected to preferentially metastasize to the brain (Valiente et al., 2020). Our original candidate, AD-2441 was able to significantly reduce clonogenic survival in MDA-MB-231BR cells, similar to control VY-3-249 (Figure 5A). Following modifications to AD-2441, we tested the analogs AD-7346, AD-5584, AD-8007, AD-1363 and AD-3766 at 100 μM where AD-5584, AD-8007, AD-1363, and AD-3766 showed significant reduction in clonogenic survival (Figure 5B). Since targeting ACSS2 blocked BCBM clonogenic cell survival, we tested whether our top candidates drugs, AD-8007 and AD-5584 could increase cell death in BCBM cells. Treatment of brain trophic cells, MDA-MB-231BR (Figure 5C) and 4T1BR (Supplementary Figure S3A) with AD-8007 and AD-5584 significantly increased cell death compared to control treatment, as measured with propidium iodide (PI) staining, and was comparable to effects seen with VY-3-135. Taken together, these data suugest that new ACSS2 inhibitors AD-5584 and AD-8007 can block colony cell survival and induce cell death in BCBM cells *in vitro*.

**FIGURE 6:**
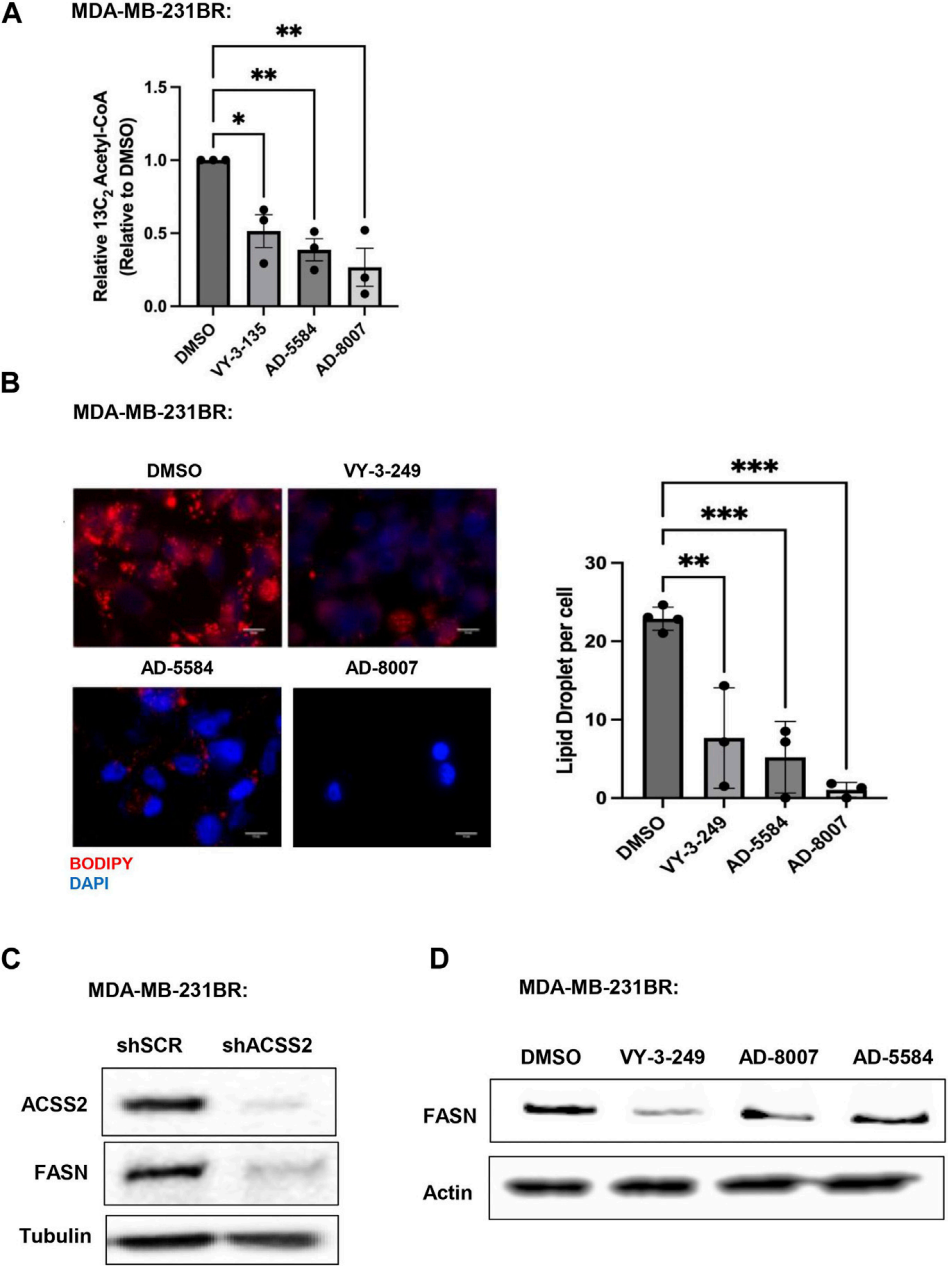
*In Vitro* effect of ACSS2 inhibitors on MDA-MB-231BR cells lipid content. **(A)** Acetyl-CoA levels were quantified by liquid chromatography-high-resolution mass spectrometry (LC-HRMS) in MDA-MB-231BR cells treated with Control (DMSO) or ACSS2 inhibitors (VY-3-135, AD-5584, AD-8007) at 100 μM for 48 h (n = 3). One-way ANOVA reported as mean ± SEM. *p-value <0.05, **p < 0.01. **(B)** Representative images of MDA-MB-231BR cells stained with BOPIDY following 48 h treatment with ACSS2 at 100 μM and presented as average lipid droplet per cell from three independent experiments (left). Quanitification of lipid content presented (right) as One-way ANOVA reported as mean ± SEM. *p < 0.05, **p < 0.01, ***p < 0.001. **(C)** Immunoblot analysis of MDA-MB-231BR cells with shRNA against scramble or ACSS2 with the indiciated antibodies. **(D)** Immunoblot analysis of MDA-MB-231BR cells treated with control (DMSO) or ACSS2 inhibitors (VY-3-249, AD-5584, AD-8007) for 24 h with the indiciated antibodies.

Since ACSS2 plays an integral role in generating acetyl-CoA from acetate, we examined the effects of novel inhibitors on acetyl-CoA content. We show that treatment of MDA-MB-231BR (Figure 6A) or 4T1BR (Supplementary Figure S3B) cells with AD-8007 and AD-5584 significantly reduced acetyl-CoA levels comparable to those seen in VY-3-135 treatment. Furthurmore, acetyl-CoA derived from ACSS2 is a vital substrate for lipid synthesis (Huang et al., 2018), leading us to examine the effect of novel ACSS2 inhibitors on lipid content. In line with the reduction of acetyl-CoA levels, we found AD-8007 and AD-5584 compounds significantly reduced lipid droplet content in MDA-MB-231BR cells when compared to control treatment (Figure 6B). Previous studies have shown fatty acid synthesis is elevated and critical for breast tumors growing in the brain and the enzyme responsible for *de novo* lipid synthesis, fatty acid synthase (FASN) is required for growth and survival of these tumors (Breast Cancer Brain Metastases Rely, 2021; Ferraro et al., 2021; Menendez and Lupu, 2022; Cheng et al., 2023). In liver cancer cells, acetate-induced lipogenesis is regulated by ACSS2 and ACSS1 and is associated with regulation of FASN expression (Gao et al., 2016). We show that genetic targeting of ACSS2 with RNAi caused reduction of FASN protein levels in BCBM cells compared to control RNAi (Figure 6C). Consistent with this data, treatment BCBM cells with ACSS2 inhibitors VY-3-249, AD-8007 and AD-5584 also reduced protein levels of FASN compared to controls (Figure 6D). Thus, targeting ACSS2 with our novel inhibitors reduces acetyl-CoA and lipids levels which is associated with reduced expression of key lipogenic enzyme FASN in BCBM cells.

### Evaluation of ACSS2 inhibitors on BCBM growth *ex vivo* and synergy with radiation

**FIGURE 7:**
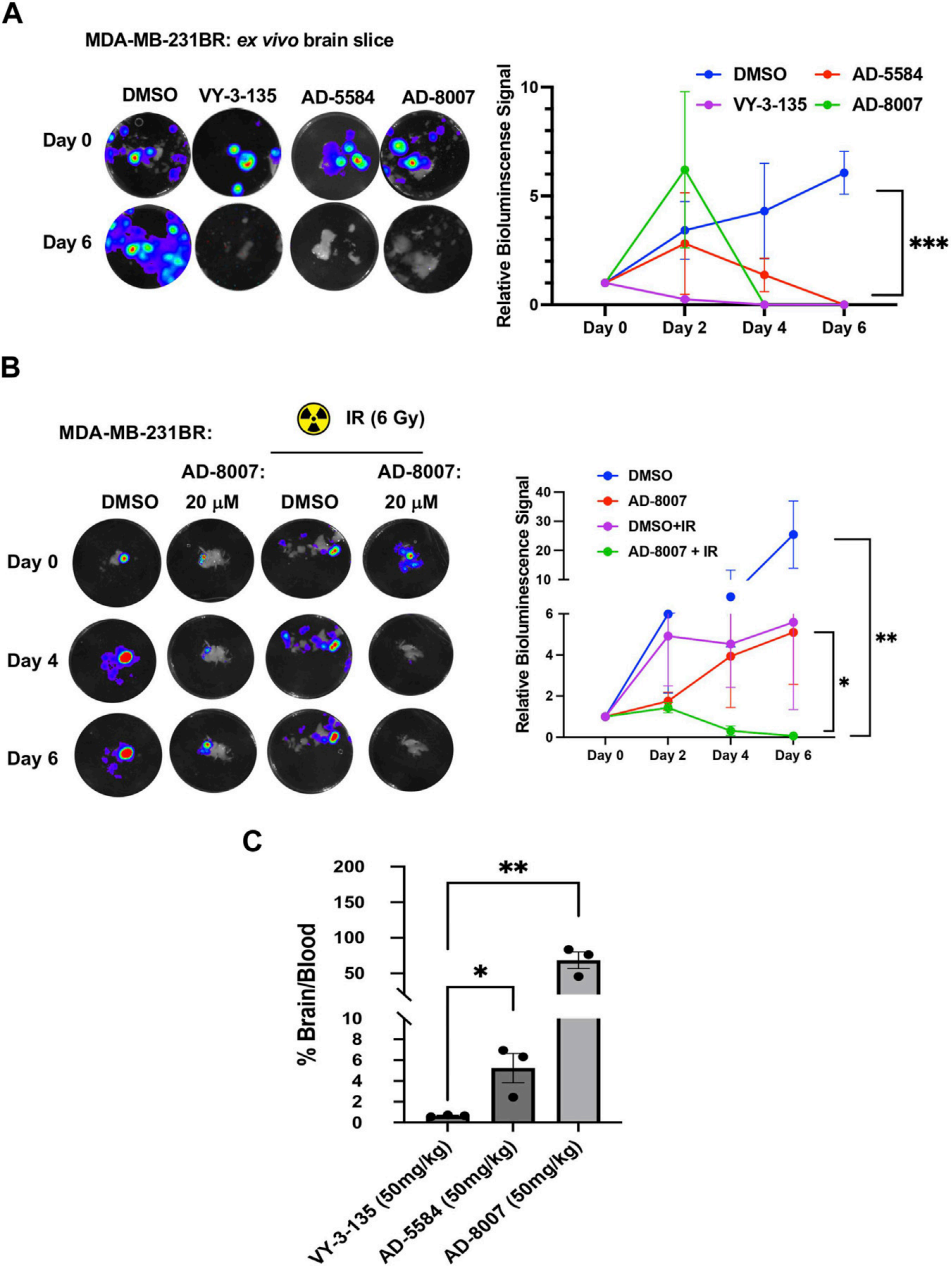
*Ex vivo* and BBB permeability of ACSS2 inhibitors. **(A)** Representative images of *ex vivo* tumor-bearing brain slices were obtained from Nu/Nu mice injected with luciferase-tagged MDA-MB-231BR cells treated with ACSS2 inhibitors at 100 μM for 6 days. Quantified graph of relative bioluminescence signal at indicated day (n = 3) Two-way ANOVA reported as mean ± SEM. *p-value ***p < 0.001 **(B)** Representative images of *ex vivo* tumor bearing brain slices derived from nu/nu mice injected with luciferase tagged MDA-MB-231BR cells exposed to no irradiation (control) or one dose of 6Gy treated with ACSS2 inhibitors at 20 μM for 6 days (n = 3). ANOVA reported as mean.*p < 0.05. **(C)** Quantification of LC-MS peaks of ACSS2 inhibitor present in the brain over blood following intraperitoneal delivery of 50 mg/kg of drug for 30 min (AD-5584) or 1 h (AD-8007, VY-3–135) (n = 3). Blood extraction via intracardiac injection, perfusion, and brain retrieval. Student’s paired *t*-test reported as mean ± SEM; *p < 0.05, **p < 0.01.

To test these compounds in a more physiologically relevant model, we employed our recently developed *ex vivo* tumor-brain slice model (Ciraku et al., 2021). Treatment of *ex vivo* MDA-MB-231BR (Figure 7A) or 4T1BR (Supplementary Figure S3C) tumor bearing brain slices with ACSS2 inhibitors AD-5584 and AD-8007 significantly reduced tumor growth of preformed tumors, suggesting induction of cell death compared to controls. Reduction of preformed tumors by our novel ACSS2 inhibitors was similar to the effects of treatment of tumor bearing brain slices with VY-3-135 (Figure 7A). Additionally, treatment of brain slices not containing tumors with ACSS2 inhibitors AD-8007 and AD-5584 did not alter cell viability compared to control treatments (Supplementary Figure S3D). Thus, these novel ACSS2 inhibitors block tumor growth and survival in the brain microenvironment while causing no overt toxicity to normal brain tissue.

Radiation is one of the first lines of treatment for patients with breast cancer brain metastases (Bailleux et al., 2021). We have previously shown that irradiation of brain slices containing MDA-MB-231BR leads to cytostatic effects *ex vivo* (Ciraku et al., 2021); thus we tested whether our new ACSS2 inhibitors could synergize with radiation in blocking BCBM growth. Treatment of preformed tumors with suboptimal dose of AD-8007 (20 µM) had little effect on BCBM cell growth *ex vivo* (Figure 7B) and, as previously shown, treatment with 6 Gy radiation alone caused a cytostatic effect on BCBM growth (Figure 7B). However, combination treatment of AD-8007 and radiation significantly reduced preformed BCBM tumors *ex vivo* (Figure 7B). Thus, these data suggests that our novel ACSS2 inhibitors may be able to synergize with irradiation to block BCBM growth and survival.

### Metabolic stability and blood-brain barrier penetration assessment of AD-5584 and AD-8007

We first evaluated the metabolic and plasma stability of AD-5584 and AD-8007 using human liver microsomes (HLMs). We included AD-3766 as a control to validate our computational pipeline’s capacity to predict metabolic stability. Consistent with our predictions (Supplementary Figure S2), experimental validation indicated that both AD-5584 (T_1/2_ of 20 min) and AD-8007 (T_1/2_ of >145 min) demonstrated significantly higher metabolic stability than the control, AD-3766 (T_1/2_ of 0.9 min) (Supplementary Table S2). These findings suggest that AD-5584 and AD-8007 are promising foundations for further optimization, including potency and BBB permeability.

To evaluate the BBB permeability of AD-5584 and AD-8007, we used the human multidrug resistance protein 1 (MDR1)-transfected Madin-Darby canine kidney (MDCK) cell line (MDR1-MDCK) BBB assay (Di et al., 2013). This assay provides the apparent permeability coefficient (Papp), efflux ratio, and % recovery to assess compound permeation. AD-5584 and AD-8007 showed moderate permeability, with AD-8007 displaying a low efflux ratio, indicating its potential to bypass P-gp substrate detection and cross the BBB (Supplementary Table S3). These results align well with our computational predictions (Supplementary Figure S1C).

Our *in vitro* and *ex vivo* results revealed the potency of AD-5584 and AD-8007 as novel ACSS2 inhibitors able to block growth, reduce acetyl-CoA, lipid content, and induce cell death in BCBM cells. However, successfully translating these results to an *in vivo* context hinges on the compounds’ ability to cross the blood-brain barrier, reach their intended target, and remain metabolically stable. Although BBB permeability increases during brain metastasis progression due to the formation of a more permeable tumor-brain barrier (TBB) (Gampa et al., 2017), leveraging therapeutics at the earliest possible stage while the BBB remains intact is a key clinical objective. Thus, optimal treatment candidates should demonstrate high BBB permeability *in vivo*. Our modeling predicted that AD-5584 and AD-8007 may be more brain-permeable compared to VY-3-135 (Supplementary Figure S1C). To test this *in vivo*, we intraperitoneally injected mice with these compounds and measured levels of drugs in plasma compared to brain homogenate. Utilizing LC-MS analysis, we found that ACSS2 inhibitors AD-5584 and AD-8007 are detected at significantly higher levels in the brain compared to VY-3-135 at 50 mg/kg dose (Figure 7C). Thus, we have successfully identified and validated AD-5584 and AD-8007 as novel ACSS2 inhibitors able to reduce BCBM growth *in vitro* and *ex vivo*, reduce lipid content in MDA-MB-231BR cells. Importantly, both compounds demonstrated strong metabolic stability and ability to penetrate the blood-brain barrier *in vivo*.

### Evaluation of AD-8007 in targeting BCBM tumor growth *in vivo*

**FIGURE 8:**
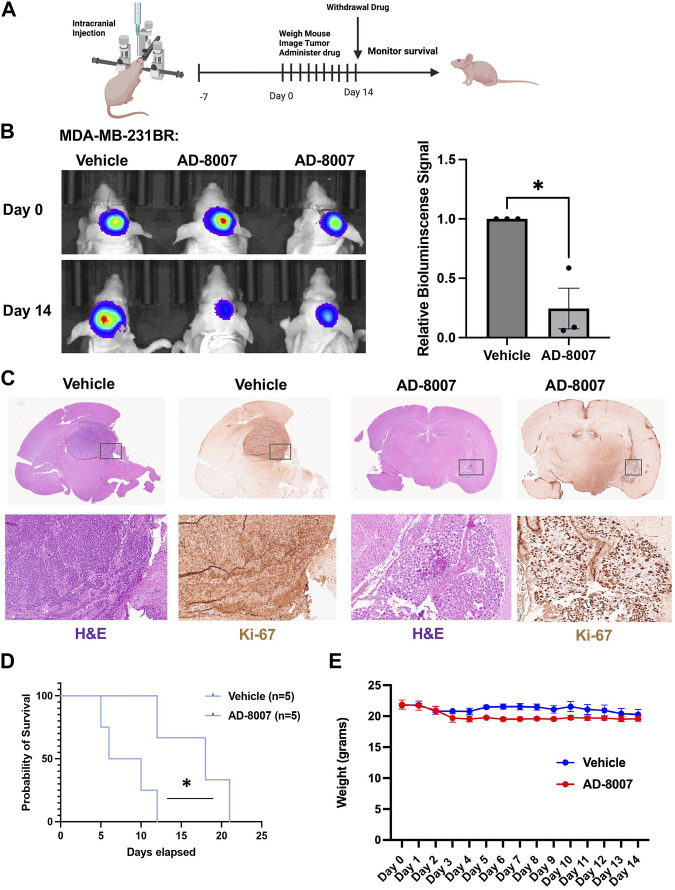
Effects of AD-8007 on BCBM growth *in vivo*. **(A)** Schematic workflow for *in vivo* studies of AD-8007 treatment. **(B)** Representative images of bioluminescent detection of tumors from Nu/Nu mice injected with luciferase tagged MDA-MB-231BR cells at Day 0 (prior to drug treatment) and at 14 days post-drug treatment. Data are quantified and presented as average Relative Bioluminescence signal from mice injected with MDA-MB-231BR cells treated with Vehicle (n = 3) or AD-8007 treated mice (n = 3) (right). Student’s t-test reported as mean ± SEM; *p < 0.05. **(C)** Representative images of brain sections stained for H&E and Ki67 at 14 days-post treatment. Top: 4x magnification. Bottom: 10x magnification. **(D)** Kaplan Meyer survival analysis quantifying survival of mice injected with MDA-MB-231BR cells and treated with vehicle (n = 5) or AD-8007 (n = 5), *p < 0.05. **(E)** Quantification of weights (grams) of mice injected with MDA-MB-231BR cells and treated with vehicle (n = 3) or AD-8007 (n = 3) for 14 days, analyzed with two-way ANOVA, n. s.

In order to test the efficacy of AD-8007 in reducing tumor burden *in vivo,* we intracranially injected luciferase tagged MDA-MB-231BR cells in an immunodeficent mice, and allowed tumor formation for 7 days. After tumor formation, we began daily administration of AD-8007 at 50 mg/kg, monitoring mouse weight and tumor burden via luciferase signal (Figure 8A). Treatment with AD-8007 significantly reduced tumor burden in MDA-MB-231BR (Figure 8B) *in vivo*. Tumors extracted immediately post-drug treatment confirm reduction in tumor burden and proliferation via H&E and Ki67 staining, respectively, in AD-8007 treated mice compared to vehicle (Figure 8C, Supplementary Figure S4B). Consistent with *in vitro* findings, treatment of mice with AD-8007 reduced expression of FASN staining in MDA-MB-231BR tumors compared to vehicle treated mice (Supplementary Figure S3E). Mice that had drug withdrawn after treatment show significantly extended surivial following AD-8007 treatment compared to vehicle in both MDA-MB-231BR (Figure 8D). Similar results were detected in immunecompetent mice contianing 4T1-BR tumors as treatment of AD-8007 reduced tumor burden (Supplementary Figure S4A), reduced Ki-67 staining (Supplementary Figure S4B), and significantly extended survival of mice (Supplementary Figure S4C). Additionally, treatment with AD-8007 did not cause significant weight lost compared to vehicle treated immunodeficient (Figure 8E) or immunecompetent mice (Supplementary Figure S4D) suggesting no apparent toxicities with AD-8007 treatment. Taken together, these data suggest treatment with AD-8007 can significantly reduce BCBM tumor burden and extend survival *in vivo*, futher validating our novel ACSS2 inhibitors as possible canidates for the treatment of BCBM patients.

## Discussion

Our study has identified and characterized novel ACSS2 inhibitors that can mitigate BCBM growth *in vitro*, *ex vivo* and *in vivo*. By applying a computational pipeline to screen and predict drug-like properties, we have identified and validated two potential compounds, AD-5584 and AD-8007, as specific inhibitors of human ACSS2. The computational pipeline effectively filtered potential ACSS2 binders from a pool of molecules. This *in silico* approach has leveraged unique computational techniques and software to predict ADME properties and other drug-like features. As *in silico* screening techniques have the potential to accelerate drug discovery and reduce costs, this study adds to the growing evidence supporting their utility.

Furthermore, our compounds AD-5584, and AD-8007, demonstrated improved drug-like characteristics and metabolic stability compared to the control compounds. This presents the promising potential for these compounds to withstand first-pass metabolism and reach their target site, essential attributes for drugs intended for systemic administration. However, it should be noted that the findings are predictive or *in vitro* validated and need further validation through *in vivo* metabolic stability assessments. In addition to identifying potential ACSS2 inhibitors, this study has also shed light on their mechanism of action. The identified compounds specifically bind directly to ACSS2, and not ACSS1, and interfere with its role in lipid metabolism, as evidenced by the reduction in lipid droplet content and induction of cell death in BCBM cells *in vitro*. Our results are consistent with studies in glioblastoma where ACSS2 plays a key role in converting acetate to acetyl-CoA and lipids (Ciraku et al., 2022). Recent studies have shown that breast tumors in the brain must adapt to the low lipid availability in the brain by increasing *de novo* fatty acid synthesis and targeting the enzyme fatty acid synthase (FASN) can block breast cancer growth in the brain (Ferraro et al., 2021). However, targeting FASN has proven problematic as many FASN inhibitors have failed to advance in the clinic due to largely unexpected *in vivo* toxicities (Qu et al., 2021). We show that in BCBM cells targeting ACSS2 reduces levels of FASN levels thus suggesting that ACSS2 may be an attractive alternative therapeutic strategy for the treatment of lipid-dependent brain tumors. Interestingly, the most potent ACSS2 inhibitors, AD-5584 and AD-8007, were found to induce cell death in BCBM cells *in vitro* but were not toxic in normal brain tissue (Supplementary Figure S3D) and did not alter weight loss in mice (Figure 8E, Supplementary Figure S4D), underscoring their potential as therapeutic candidates for BCBM. Future studies will further explore the possible toxicities of these compounds.

In assessing the BBB penetration potential of these compounds, the study has also factored in the physiological context of drug delivery, particularly considering the crucial role of BBB permeability for drugs intended to act on brain targets. The BBB is a major hurdle for delivering many drugs to the brain, and it can become more permeable during brain metastasis progression (Do et al., 2014). While BBB permeability can be leveraged during this stage of progression, for optimal treatment efficacy, drugs should be able to penetrate the BBB at the earliest stage possible. In this context, the BBB permeability of AD-5584 and AD-8007 holds promise. In addition, we show that AD-8007 can synergize with radiation treatment to block tumor growth *ex vivo*. Radiation treatment of BCBM often leads to cytostatic effects in patients (Bailleux et al., 2021) that we have shown can be modeled *ex vivo* (Ciraku et al., 2021) (Figure 7B). Future studies will focus on evaluating tumor growth inhibition and induction of cell death *in vivo* to strengthen the potential of these compounds as effective and well-tolerated therapeutic candidates against BCBM in synergy with radiation *in vivo*.

In conclusion, we have identified brain-penetrant ACSS2 inhibitors that can mitigate BCBM growth. Treating breast cancer patients that have macrometastasis in the brain remains a major clinical challenge as there are few therapeutic options and median overall survival for these patients is measured in months (Watase et al., 2021). Our study, for the first time, shows that treating mice that contain breast cancer brain metastatic macrometastasis with an ACSS2 inhibitor is effective in shrinking tumors in the brain parenchyma and also can extend survival of these mice. It is not clear whether these inhibitors will block other aspects of the metastatic casacade including cellular invasion, anoikis resistance, extravasation, and others. Future studies will address these additional possible effects. Nevetheless, the potential of the identified inhibitors, AD-5584 and AD-8007, their impact on cell growth and lipid metabolism, along with their high BBB permeability and metabolic stability, present encouraging avenues for further investigation and development as potential therapeutic candidates for targeting breast cancer tumors in the brain. We aim to further optimize our hit compounds to reach a clinically relevant range, focusing on increasing metabolic stability and potency of AD-5584 and AD-8007, further developing these drugs for treating patients with cancer brain metastases.

## Significance

This work aims to provide new brain-penetrant small molecules targeting a metabolic vulnerability of brain growing cancers, including breast cancer brain metastatic tumors. This article focuses on targeting the metabolic enzyme ACSS2, which has been implicated as a key regulator of tumor growth in the brain. Brain growing tumors use acetate via ACSS2 to generate acetyl-CoA and drive *de novo* lipogenesis, representing a targetable metabolic vulnerability. Indeed, we show that our new brain-penetrant inhibitors AD-5584 and AD-8007 reduce lipid levels and tumor cell survival. These finding are of high impact as they show the utility of brain-penetrant ACSS2 inhibitors in treating breast cancer brain metastases and possible synergy with radiation.

## Experimental model and study participant details

### Cell culture

Triple-negative brain trophic cells MDA-MB-231BR and 4T1BR cells were a kind gift from Dr. Patricia Steeg (Center for Cancer Research, National Cancer Institute) (Achrol et al., 2019). Both cell lines were cultured in DMEM supplemented with 10% fetal bovine serum (FBS), 5% 10,000 Units/mL Penicillin-10,000 μg/mL Streptomycin, and 5% 200 mM L-Glutamine. For crystal violet staining, 5 × 10^4^ cells were plated and subjected to the treatments as described in the individual figures and then stained with 0.5% crystal violet prepared in a 1:1 methanol-water solution followed by PBS washes. ACSS2 inhibitors were dissolved in 100% ultra-pure DMSO.

### Animal experiments

Intracranial injections were performed as previously described (Ciraku et al., 2021). Briefly, Nu/Nu or BalbC female 4–6 week old mice from Charles River Laboratories (Wilmington, MA, United States) were immobilized using the Just for Mice ™ Stereotaxic Frame (Harvard Apparatus, Holliston, MA, United States) and injected intracranially with 5 μL of a 20,000 cells/μL solution of MDA-MB-231BR or 4T1BR cells stably expressing luciferase. Mice were injected intraperitoneally with 30 mg/kg of Dluciferin solution (Caliper Life Sciences, Hopkinton, MA) and results analyzed using Living Image software (Caliper Life Sciences, Waltham, MA, United States). For *in vivo* analog trials, mice were weighed daily and injected intraperitoneally with 50 mg/kg of AD-8007. Mice were euthanized 3 weeks after injection following survivial or at the end of drug treatment at indiciated day. The animal study was reviewed and approved by the Institutional Animal Care and Use Committee.

### *Ex vivo* brain slice model

*Ex vivo* brain slice tumor model was performed as previously described (Ciraku et al., 2021). Briefly, Nu/Nu athymic 4–6 week old mice Charles River Laboratories (Wilmington, MA, United States) were immobilized using the Just for Mice Stereotaxic Frame (Harvard Apparatus, Holliston, MA, United States) and injected intracranially with 5uL of 20,000 cells/uL solution of luciferase tagged MDA-MB-231BR or 4T1BR cells. Tumor growth was monitored via bioluminescence imaging on the IVIS system (Perkin Elmer, Waltman, MA, United States). Organotypic hippocampal cultures were prepared as described previously (Ciraku et al., 2021). Briefly, adult mice (4–6 weeks) or mice after 12 days following intracranial injection were decapitated and their brains rapidly removed into ice-cold (4°C) sucrose-aCSF composed of the following (in mM): 280 sucrose, 5 KCl, 2 MgCl_2_, 1 CaCl_2_, 20 glucose, 10 HEPES, 5 Na^+^-ascorbate, 3 thiourea, 2 Na^+^-pyruvate; pH = 7.3. Brains were blocked with a sharp scalpel and sliced into 250 µm sections using a McIlwain-type tissue chopper (Vibrotome Inc.). Four to six slices were placed onto each 0.4 µm Millicell tissue culture insert (Millipore) in six-well plates, 1 mL of medium containing the following: Neurobasal medium A (Gibco), 2% B27 supplement (Gibco), 1% N2 supplement (Gibco), 1% glutamine (Invitrogen), 0.5% glucose, 10 U/mL penicillin, and 100 ug/mL streptomycin (Invitrogen), placed underneath each insert. The media was changed every 2 days following imaging. Tumor growth was monitored via bioluminescence imaging on the IVIS 200 system (Perkin Elmer), and results were analyzed using Living Image software (Caliper Life Sciences, Waltham, MA, United StatesUnited States). For irradiation studies, the *ex vivo* tumor-brain slices were irridated prior to drug treatment with a single dose of 6 Gy (310 kVp x-rays), as previously described (Ciraku et al., 2021). For the MTS assay, individual brain slices were transferred to a 96-well plate and subjected to Promega CellTiter 96^®^ Aqueous One Solution (Cat: G3582) mixed in a 1:5 ratio with culture media and treated as previously described (Ciraku et al., 2021). Tissues were incubated at 37 ℃ , 5% CO_2_ for 4 h, and absorbance at 490 nm was measured with a Tecan Spark Microplate reader.

## Method details

### Production of lentivirus and viral transduction

HEK-293T cells were grown to ∼90% confluency and transfected. Prior to transfection, 20 μg of shRNA or overexpression plasmid DNA, 10 μg VSVG, 5 μg RSV, and 5 μg RRE were gently mixed and incubated in 1.5 mL of Optimem for 5 min. Concurrently, 105 μL of PEI was added dropwise to 1.5 mL of Optimem and incubated for 5 min. Following the 5 min incubation, the PEI solution was added dropwise to the DNA solution and incubated for at least 30 min. The PEI-DNA solution was then added dropwise to the HEK-293T cells already plated with 5 mL of Optimem and the cells were incubated overnight in the transfection media. Approximately 16–18 h later, the transfection media was replaced with normal growth media. Viral supernatants were collected at 24 and 48 h following the media change. These supernatants were passed through a 0.45 μm filter and portioned into 1 mL aliquots to be stored at −80°C if not for immediate use. 1 mL aliquots from each collection time point where mixed with 2 mL of growth media and 1:500 8 mg/mL polybrene and added to 75% confluent cell line of interest for 6 h and replaced with 10 mL of growth media followed by the appropriate antibiotic selection.

### RNA interference

Control shRNA was acquired from Addgene (plasmid 1864), from D. Sabatini (Massachusetts Institute of Technology). Control-scrambled shRNA sequence used was: CCT​AAG​GTT​AAG​TCG​CCC​TCG​CTC​TAG​CGA​GGG​CGA​CTT​AAC​CTT. ACSS2 shRNA used: ACSS2 shRNA sequence used (Sigma): CCG​GGC​TTC​TGT​TCT​GGG​TCT​GAA​TCT​CGA​GAT​TCA​GAC​CCA​GAA​CAG​AAG​CTT​TTT G.

### Reagents

Anti-actin (Santa Cruz Biotechnology; Dallas, TX, United States), Anti-ACSS2, Anti-FasN,Anti-beta Tubulin (Cell Signaling Technology; Danvers, MA, United States). Puromycin, Polybrene crystal violet (Sigma Aldrich, St. Louis, MO, United States), D-luciferin potassium salt (Perkin Elmer, Waltham, MA, United States).

### Immunohistochemistry

Brains were dissected, fixed in 4% formalin and prepared for processing/sectioning/embedding/blocking to generate paraffin embedded slides. The slides containing brain metastatic tumors were deparaffinized by Xylene and subsequent rehydration was done by decreasing concentrations of ethanol-water mixture. Antigen retrieval was done by citrate buffer immersion and steaming the slides for 45 min. Tissue was treated with 200–400 ul 1% BSA+5% serum PBS solution for 1h. Primary incubation was done at 4^o^ C overnight using primary antibody anti-FasN (Cell Signaling Technologies, C20G5). Secondary antibody incubation was done for an hour at RT with HRP conjugated Anti-Rabbit Secondary antibody (Cell Signaling Technologies). at 1:100 dilution. For IHC the stain was developed using the DAB kit by Vectastain (Vector Labs, Burlingame, CA, United States). Finally, slides were mounted and imaged under a light microscope. H&E and Ki-67 staining were performed and assessed at TJU, Department of Pathology, Philadelphia, PA by board-certified pathologist.

### Immunoblotting

Immunoblotting protocols have been previously described. Briefly, cell lysates from one to five x 10^6^ cells were prepared in radioimmune precipitation assay buffer (150 mM NaCl, 1% NP40, 0.5% DOC, 50 mM Tris HCL at pH 8, 0.1% SDS, 10% glycerol, 5 mM EDTA, 20 mM NaF, and 1 mM Na_3_VO_4_) supplemented with 1 μg/mL each of pepstatin, leupeptin, aprotinin, and 200 μg/mL PMSF. Lysates were cleared by centrifugation at 16,000 x *g* for 15 min at 4°C and analyzed by SDS-PAGE and autoradiography with chemiluminescence. Proteins were analyzed by immunoblotting using primary antibodies indicated above.

### Clonogenic survival assay

To assess clonogenic survival, 1 × 10^5^ cells were seeded into a 6-well plate until 70%–80% confluency and treated for 48 h with analogs at 100 uM (unless otherwise stated in the figure legend). After 48 h, cells were trypsinized and counted using hemocytomer, and 1 × 10^4^ cells per treatment were plated into a 6-well plate with fresh culture medium and allowed to grow for 10 days. Following 10-day incubation, cells were washed twice with PBS, stained with crystal violet for 30 min, and washed with dH_2_O twice. Colonies >50mircons were counted. For crystal violet staining, 0.5% crystal violet was prepared in a 1:1 methanol-water solution.

### BODIPY staining of cells

Cells were treated with 5uM BODIPY 493/503 (Invitrogen) and NucBlue^®^ Live reagent (Invitrogen) in PBS for 15 min, washed 2x with 1xPBS, fixed using 4% paraformaldehyde for 30 min at RT in the dark and washed in 1xPBS prior to mounting and imaging on EVOS FL (Life Technologies) using Texas Red filter for BODIPY and DAPI channel for NucBlue.

### Flow cytometry

Cells were prepared according to manufacturer protocol (BD Pharmingen, Propidium Iodine). Briefly, cells were trypsinized (0.25% Trypsin), counted, washed twice with 1xPBS, and resuspended in 100uL 1X binding buffer incubated with 5 uL Propidium Iodine (PI) staining solution for 15 min in the dark at room temperature. Following incubation, the volume was brought up to 500uL of 1X binding buffer. Tubes were then analyzed using a Guava easyCyte flow cytometer. All data were collected and analyzed using a Guava EasyCyte Plus system and CytoSoft (version 5.3) software (Millipore). Data are gated and expressed relative to the appropriate unstained and single-stained controls.

### BBB permeablity *in vivo*

Human ACSS2 inhibitors were prepared in 10 mg/mL saline solution. BalbC 4–6 week-old mice were weighed and injected with 50 mg/kg or 100 mg/kg of drug saline solution via intraperitoneal injection. After 30 min or 60 min, mice treated with AD-5584 or AD-8007 and VY-3-135 were placed in isoflurane, and blood was extracted and perfused via intracardiac injection. Following perfusion, mice were decapitated, and their brains were rapidly removed into ice-cold PBS. For analysis of blood samples, 200 µL of blood was transferred and allowed to clot for 15 min at room temperature. Samples were precipitated at 16000xg for 1 min, and 50 µL of serum was transferred. To serum, 200 µL of methanol was added, and samples were centrifuged at 16000 *g* for 5 min. The supernatant was analyzed by LCMS. For analysis of brain samples, brains were homogenized via bead beating using four 2.3 mm stainless steel beads in 1 mL of MeOH and 0.5 mL of H_2_O. After centrifugation for 15 min at 3000xg, the supernatant was analyzed by LCMS. LCMS was performed on an Acquity I-Class UPLC system coupled to a Synapt G2Si HDMS mass spectrometer in positive ion mode with a heated electrospray ionization (ESI) source in a Z-spray configuration. LC separation was performed on a Waters Acquity UPLC BEH 1.7 μm 2.1 × 50 mm column equipped with a Vanguard guard column, using a 0.6 mL/min solvent flow of A/B 95/5 to 15/85 in 4 min, followed by washing and reconditioning the column. Eluent A is 0.1% v/v formic acid in water, and B is 0.1% v/v formic acid in acetonitrile. Conditions on the mass spectrometer were as follows: capillary voltage 0.5 kV, sampling cone 40, source offset 80, source 120°C, desolvation temperature 250°C, cone gas 0, desolvation gas 1000 L/h and nebulizer 6.5 bar. The analyzer was operated in resolution mode. Low energy was collected between 100 and 1,500 Da at 0.2 s scan time. MSe data was collected using a ramp trap collision energy 20–40 V. Masses were extracted from the TOF MS TICs using an abs width of 0.05 Da. Data was analyzed using Waters MassLynx and Waters Unifi. Calibration curves of authentic standards were used for quantification.

### Overproduction and purification of human ACSS2

Overproduction of ACSS2 was achieved using a prokaryotic expression system. Briefly, the plasmids containing the C- and N-terminally His-tagged human ACSS2 DNA were transformed into BL21 (DE3) RIL competent cells (Agilent Technologies, Wilmington, DE) and were expressed in auto-inducing media ZYP-5052 overnight at 15°C with shaking at 225 rpm. The bacterial expressions were then spun down, the supernatant discarded, and the pellets resuspended in 50 mM Tris HCl pH8.0, 800 mM NaCl, 5 mM Imidazole, 2 mM MgCl_2_, 10% Glycerol, 0.1 mg/mL Lysozyme, 1 mM Protease inhibitor (PMSF). After the cells were lysed via sonication, the sample was subjected to ultracentrifugation, and the clarified lysate was applied to a 5 mL Talon cobalt resin affinity column (Clonetech Laboratories, Mountain View, CA). The bound protein was washed with 300 mL wash buffer 1 (20 mM Tris HCl pH8.0, 800 mM NaCl, 15 mM Imidazole, 5 mM MgCl_2_, 10% Glycerol, 0.1% CHAPS) and 300 mL wash buffer 2 (20 mM Tris HCl pH8.0, 400 mM NaCl, 18 mM Imidazole, 5 mM MgCl_2_, 10% Glycerol) prior elution with 20 mM Tris HCl pH8.0, 400 mM NaCl, 300 mM Imidazole, 2 mM MgCl_2_, 5% Glycerol. Eluted ACSS2 was then dialyzed overnight into 20 mM Tris HCl pH8.0, 150 mM NaCl, 5% Glycerol, concentrated and applied to a Superdex S-200 (16/600) using the same buffer without Glycerol. ACSS2 fractions were pooled, concentrated to 3 mg/mL, aliquoted, and stored at −80°C. hACSS1 was obtained from a commercial vendor (www.mybiosource.com).

### SPR characterization

All binding assays were performed on a ProteOn XPR36 SPR Protein Interaction Array System (Bio-Rad Laboratories, Hercules, CA, United States). The instrument temperature was set at 25°C for all kinetic analyses. ProteOn GLH sensor chips were preconditioned with two short pulses each (10 s) of 50 mM NaOH, 100 mM HCl, and 0.5% sodium dodecyl sulfide. Then, the system was equilibrated with running buffer (1x PBS pH 7.4, 3% DMSO, and 0.005% polysorbate 20). The surface of a GLH sensor chip was activated with a 1:100 dilution of a 1:1 mixture of 1-ethyl-3-(3-dimethylaminopropyl) carbodiimide hydrochloride (0.2 M) and sulfo-N-hydroxysuccinimide (0.05 M). Immediately after chip activation, the human ACSS2 or hACSS1 proteins were prepared at a concentration of 10 μg/mL in 10 mM sodium acetate, pH 5.5, and injected across ligand flow channels for 5 min at a flow rate of 30 μL/min. Then, after unreacted protein had been washed out, excess active ester groups on the sensor surface were capped by a 5 min injection of 1M ethanolamine HCl (pH 8.0) at a flow rate of 5 μL/min. A reference surface was similarly created by immobilizing a nonspecific protein (IgG b12 anti-HIV-1 gp120; was obtained through the NIH AIDS Reagent Program, Division of AIDS, NIAID, NIH: Anti-HIV-1 gp120 Monoclonal (IgG1 b12) from Dr. Dennis Burton and Carlos Barbas) and was used as a background to correct nonspecific binding. Serial dilutions of ACSS2 inhibitors or a single concentration at 25 mM of AD-5584, AD-8007, and VY-3-249 for hACSS1 binding were then prepared in the running buffer and injected at a flow rate of 100 μL/min for a 50 s association phase, followed by up to a 5 min dissociation phase using the “one-shot kinetics” capability of the ProteOn instrument. Data were analyzed using the ProteOn Manager Software version 3.0 (Bio-Rad). The responses from the reference flow cell were subtracted to account for the nonspecific binding and injection artifacts. Experimental data were fitted to a simple 1:1 binding model. Experiments were performed in triplicate to detect kinetic and equilibrium dissociation constants (K_D_).

### Fluorescence polarization-based ACSS2 biochemical assay (ATP to AMP conversion)

ACSS2 enzyme activity was measured using the TranScreener AMP^2^/GMP^2^ Assay Kit—FP Readout assay (BellBrook Labs). The assay was performed in white, opaque, 96-well plates. Compounds diluted in 100% DMSO were used starting at 150 nM with a 1:3 dilution, and ACSS2 was used at 100 nM. ACSS2 was used in assay buffer (30 mM HEPES, pH 7.4, 140 mM NaCl, 2 mM MgCl_2_, 5 mM sodium acetate, 2 mM DTT, 0.05% CHAPS). Substrate mix was added, followed by a 60-min incubation. Final substrate concentrations were 5 mM acetate, 50 μM ATP, and 5 μM CoA. After incubation, conjugated AMP antibody and AMP tracer were added according to the methods described by BellBrook Labs. After 30 min, the FP signal was measured using a Tecan Spark multimode microplate reader. In the analysis, data were normalized to represent the percentage inhibition of ATPase activity. A value of 100% inhibition corresponded to the counts observed in the absence of ACSS2, whereas 0% inhibition was aligned with the counts from the complete reaction, including a DMSO control.

### Pharmacophore-based shape screen and AD-2441 analog identification (HT screening)

The reference Quinoxaline molecule (Comerford et al., 2014) was drawn and prepared in VIDA 5.0.4.0 (OpenEye, Cadence Molecular Sciences, Santa Fe, NM. http://www.eyesopen.com) and then exported to Szybki 2.6.0.1 (OpenEye, Cadence Molecular Sciences, Santa Fe, NM. http://www.eyesopen.com) for in solution minimization to be used as the lowest possible conformer. The ChemBridge diversity library was downloaded from their database website (https://chembridge.com) and prepared by Szybki 2.6.0.1 (OpenEye, Cadence Molecular Sciences, Santa Fe, NM. http://www.eyesopen.com). Then, about 200 conformers were generated for each molecule in the library using Omega Szybki 4.2.2.1 (Hawkins et al., 2010) (OpenEye, Cadence Molecular Sciences, Santa Fe, NM. http://www.eyesopen.com). ROCS Szybki 3.6.0.1 (Hawkins et al., 2007) (OpenEye, Cadence Molecular Sciences, Santa Fe, NM. http://www.eyesopen.com) was then used to build the 3D query for the reference Quinoxaline molecule, and then we screened the prepared ChemBridge library conformers against the query. The step was repeated twice to validate the hits and select the top 5,000 hits. We then used StarDrop V7.3 (Optibrium Ltd. Cambridge, United Kingdom) to predict the drug-like properties (using the CNS penetration module) for preselection. To facilitate and improve confidence for hit selection, we continued with a structure-based docking approach, including predicting binding affinity (using the HYdrogen Bond and DEhydration Energies (HYDE) function) (Schneider et al., 2013) in SeeSAR 12.1 (BioSolveIT Gmbh, Sankt Augustin, Germany) and the homology model (swissmodel.expasy.org) of ACSS2 based on the crystal structure of ACSS2 from *Salmonella enterica* (PDB: 5JRH) which was prepared using Flare, version 5 (Cresset^®^, Litlington, Cambridgeshire, United Kingdom, http://www.cresset-group.com/flare/) to select the top 30 molecules for experimental evaluation.

After we evaluated the first 30 hits and identified AD-2441, we used two approaches for analog identification. The first approach included a simple Tanimoto coefficient cutoff of >0.9 within the ChemBridge.com library with subsequent SeeSAR 12.1 HYDE binding affinity evaluation and StarDrop V7.3 for drug-like properties filtering. The second approach included a ligand-focused SAR approach using a field-based search within Forge V10.6 (Cresset^®^, Litlington, Cambridgeshire, United Kingdom) and the Diversity library from Chembridge, followed by SeeSAR 12.1 HYDE binding affinity evaluation and StarDrop V7.3 for drug-like properties filtering.

### Docking calculations for ACSS2 inhibitors (figure preparation)

The ACSS2 inhibitors were prepared, and then energy minimized using Flare version 5 (Cresset^®^, Litlington, Cambridgeshire, United Kingdom, http://www.cresset-group.com/flare/) with a root mean squared (RMS) gradient cutoff of 0.2 kcal/mol/A and 10,000 iterations. The homology model of ACSS2 based on the crystal structure of ACSS2 from *Salmonella enterica* (PDB: 5JRH) was prepared using Flare, version 5 (Cresset^®^, Litlington, Cambridgeshire, United Kingdom, http://www.cresset-group.com/flare/) to allow protonation at pH 7.0 and removal of residue gaps. Docking calculations were performed using DiffDock (Corso et al., 2022) (github.com/gcorso/DiffDock), a diffusion generative model, using a blind docking approach (grid box over the entire protein). During the docking procedure, we used 100 inference steps and 300 samples per complex with a batch size of 12. Based on the DiffDock confidence score and SMINA score, the best 3 complexes were chosen. While DiffDock provides exceptional accuracy for blind docking, we noticed difficulties in the quality of the final poses. Therefore, we used Flare, version 5, to energy minimize the best complex form DiffDock, using the accurate XED force field minimization algorithm with a gradient cutoff of 0.050 kcal/mol/A and 10,000 iterations. In order to validate the quality of our homology model and DiffDock docking approach, we docked Adenosine-5′-propylphosphate (extracted from PDB: 1PG4) and compared the pose to the crystal structure (PDB: 1PG4).

### Acetyl-CoA quantification

Cells were grown to ∼80% confluency in a 10-cm dish in normal growth medium, 48 h prior to collecting cells, aspirate media and replace with fresh growth media and drug was added at 100 μM. To collect cells, media was aspirated and 1 mL of 10% trichloroacetic acid (w/v) in water was added directly to the cells. Cells were scraped and transferred into a 1.7 mL Eppendorf tube and stored at −80 until labeling. Acyl-CoAs were quantified by liquid chromatography-high-resolution mass spectrometry (LC-HRMS) as previously described (Kantner et al., 2024). Short-chain ^13^C_3_,^15^N_1_-acyl-CoA internal standards (ISTD) were generated in yeast as previously described (Snyder et al., 2015). For sample analysis, 50 µL of short-chain acyl-CoA ISTD was added to the sample and then cell suspensions were sonicated with 5 × 0.5-s pulses at 50% intensity (Fisherbrand™ Sonic Dismembrator Model 120 with Qsonica CL-18 sonicator probe). Lysates were centrifuged 17,000 *g* for 10 min at 4°C and supernatant was transferred to a deep-well 96-well plate for loading in a Tomtec Quadra4 liquid handling workstation. On the liquid handling workstation, lysates were applied to an Oasis HLB 96-well elution plate (30 mg of sorbent per well) pre-conditioned and equilibrated with 1 mL of methanol and 1 mL of water, respectively. After de-salting with 1 mL of water, analytes were eluted into a deep-well 96-well plate using 1 mL of 25 mM ammonium acetate in methanol. Eluent was evaporated dried under nitrogen gas. The dried LC-HRMS samples were resuspended in 50 µL of 5% (w/v) sulfosalicylic acid in water. 5 μL injections of each sample were analyzed using an Ultimate 3,000 quaternary ultra-high performance liquid chromatograph coupled with a Q Exactive Plus mass spectrometer (Thermo Scientific) as previously described (Frey et al., 2016). A modified gradient using solvent A (5 mM ammonium acetate in water), solvent B (5 mM ammonium acetate in 95:5 (v:v) acetonitrile: water) and solvent C (0.1% (v/v) formic acid in 80:20 (v:v) acetonitrile: water). Data was acquired using XCalibur 4.0 (Thermo Scientific) and analyzed using Tracefinder 5.1 (Thermo Scientific).

### Statistical analysis

All results shown are results of at least three independent experiments and are shown as averages and presented as mean ± s. e (or SEM if stated). *p*-values were calculated using a Student’s two-tailed test (* represents *p*-value ≤0.05 or **p-value ≤0.01 or as marked in figure legend). Statistical analysis of the growth rate of mice was performed using ANOVA. *p-value <0.05.

## Data Availability

The original contributions presented in the study are included in the article/Supplementary Material; further inquiries can be directed to the corresponding authors.

## Supplementary material

SUPPLEMENTARY FIGURE S1 Computational prediction of drug-like properties of AD-2441 and its analogs. **(A)** P-glycoprotein (Pgp) category and predicted probability of AD-2441 and analogs. **(B)** Predicted plasma protein binding (90% threshold, PPB90) category and predicted probability. **(C)** Predicted blood-brain barrier (BBB) distribution/category and category-probability of AD-2441 and analogs.

Supplementary Figure S2 Computational prediction of metabolic stability of AD-2441 and its analogs. **(A)** Prediction of the major metabolizing CYP isoforms for AD-2441 and analogs. The majority of compounds are predicted to be metabolized by the 3A4 isoform, except AD-1363, which is predicted to be metabolized by the 2D6 isoform. **(B)** A lower boundary for predicted 3A4 affinity of AD-2441 and analogs using the hydrogen bond and dehydration scoring function (HYDE) implemented in SeeSAR12.1. 2D6 affinity category of AD-2441 and analogs as predicted by the Stardrop P450 module. **(C)** Overall composite site lability (CSL) score and number of labile sites (for metabolism) for AD-2441 and analogs. A lower CSL score indicates a more stable molecule. The prediction was achieved using the StarDrop (version 7) P450 module. **(D)** SPR-derived Response Units (RU) at 25μM injection of VY-3-249, AD 5584, and AD-8007 to immobilized human ACSS2 or human ACSS1. Experiments were performed in triplicate (n=3), and statistical significance was performed using paired comparison and means compared using the Tukey method. Boxblots display the mean (line in the box and value to the right), box size represents SEM, and whiskers the confidence interval at α of 90%.

SUPPLEMENTARY FIGURE S3 Effect of ACSS2 inhibitors on 4T1BR cells in vitro and ex vivo.** (A)** Quantification of cell death as measured by Propidum Idodine (PI)+ cells detected by flow cytometry analysis of 4T1BR cells treated with ACSS2 inhibitors at 100 μM for 48 hours (n=3). One-way ANOVA reported as mean ± SEM; ****p<0.0001. **(B)** Acetyl-CoA levels were quantified by liquid chromatography-high-resolution mass spectrometry (LC-HRMS) in 4T1BR cells treated with Control (DMSO) or ACSS2 inhibitors (VY-3-249, VY-3-135, AD-5584, AD-8007) at 100 μM for 48 hours (n=3). One-way ANOVA reported as mean ± SEM; ****p<0.0001 **(C)** Representative images of ex vivo tumor-bearing brain slices were obtained from nu/nu mice injected with luciferase-tagged 4T1BR cells treated with ACSS2 inhibitors at 100 μM for 6 days. Quantified graph of relative bioluminescence signal at indicated day (n=3). Two-way ANOVA reported as mean ± SEM; *p<0.05. **(D)** Ex vivo brain slices were treated as in HYPERLINK ”#fig7“ Figure 7A (without tumor) with either DMSO or 100 =M of AD-8007 or AD-5584, collected on day 6, and analyzed for cell viability (MTS assay). As a positive control, slices were treated with paraformaldehyde (PFA) for 2 hrs, rendering the brain slices non-viable (n=3). Student’s t-test reported as mean ± SEM; *p<0.05. **(E)** Immunohistochemical staining on coronal sections from brains inoculated with MDA-MB-231BR cells and treated with vehicle or AD-8007 for 14 days. Top: H&E staining (10X magnification), bottom: FASN staining (10X magnification).

SUPPLEMENTARY FIGURE S4 Effects of ACSS2 inhibitors on 4T1BR cells in vivo. **(A)** Representative images of bioluminescent detection of tumors from BalbC mice injected with luciferase tagged 4T1BR cells at Day 0 (prior to drug treatment) and at 10 days post-drug treatment. **(B)** Representative images of brain sections stained for H&E and Ki67 at 10 days-post treatment. Data are quantified and presented as average Relative Bioluminescence signal from mice injected with 4T1BR cells treated with Vehicle (n=4) or AD-8007 treated mice mice (n=4) (right). Student’s t-test reported as mean ± SEM; ***p<0.001. **(C)** Kaplan Meyer survival analysis quantifying survival of mice injected with 4T1BR cells and treated with vehicle (n=4) or AD-8007 (n=4), *p<0.05. **(D)** Quantification of weights (grams) of mice injected with 4T1BR cells and treated with vehicle (n=4) or AD-8007 (n=4) for 10 days, analyzed with two-way ANOVA, n.s. SMARTQC
