# Supplementary figures and images for: Selective and brain-penetrant ACSS2 inhibitors target breast cancer brain metastatic cells
Source: Front Pharmacol. 2024 May 16;15:1394685. doi: 10.3389/fphar.2024.1394685 (PMC11137182; doi:10.3389/fphar.2024.1394685)

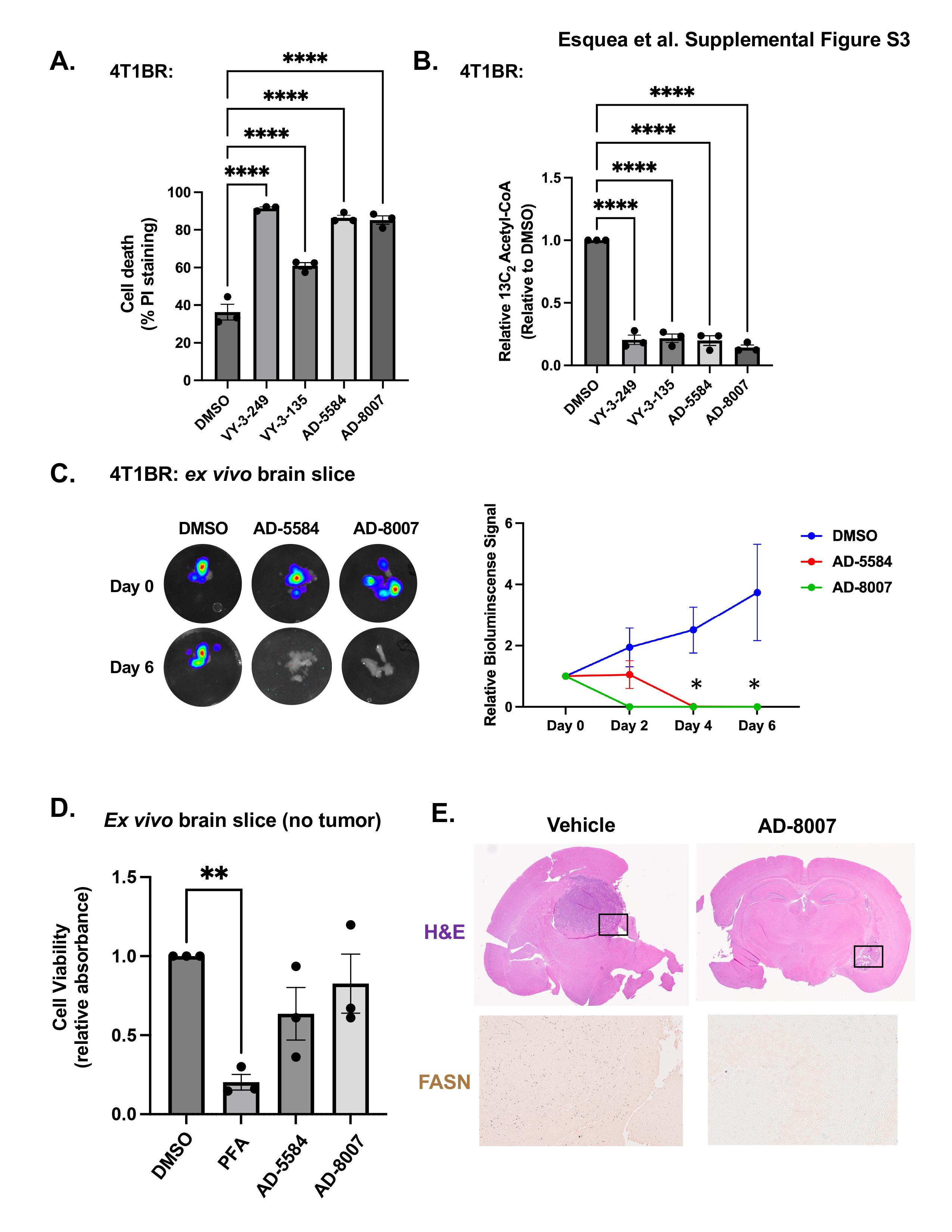

Supplement: Supplementary file 1 [file Image3.JPEG]

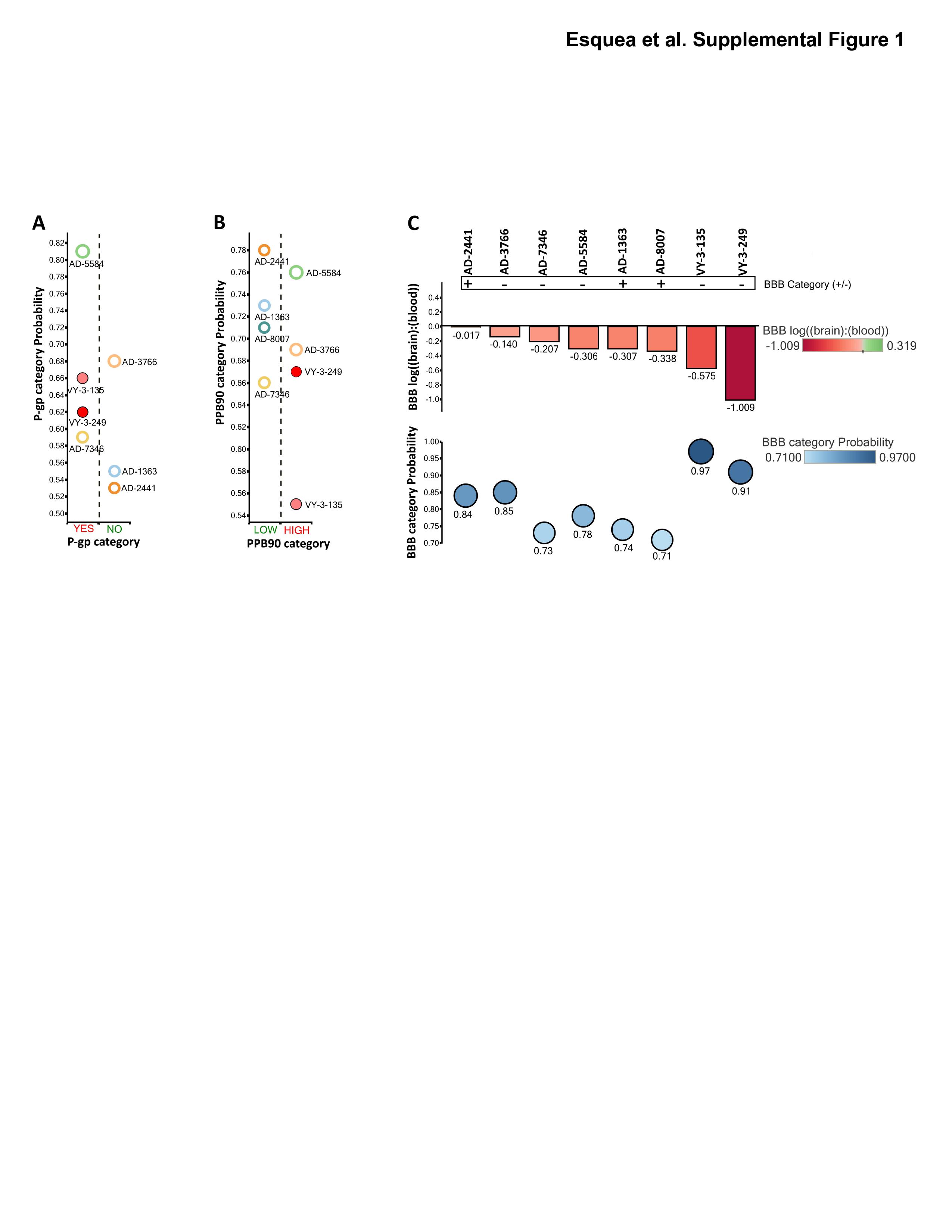

Supplement: Supplementary file 3 [file Image1.JPEG]

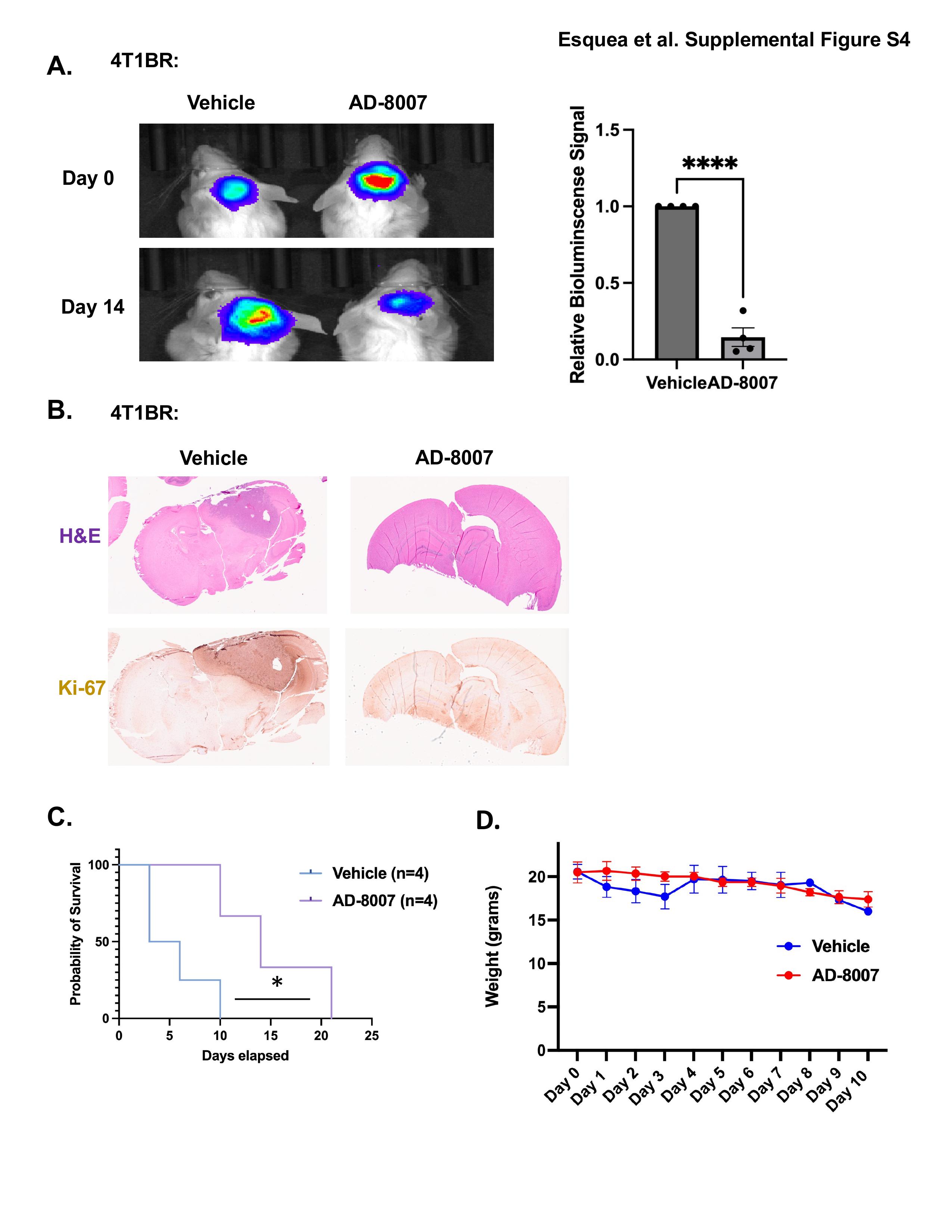

Supplement: Supplementary file 4 [file Image4.JPEG]

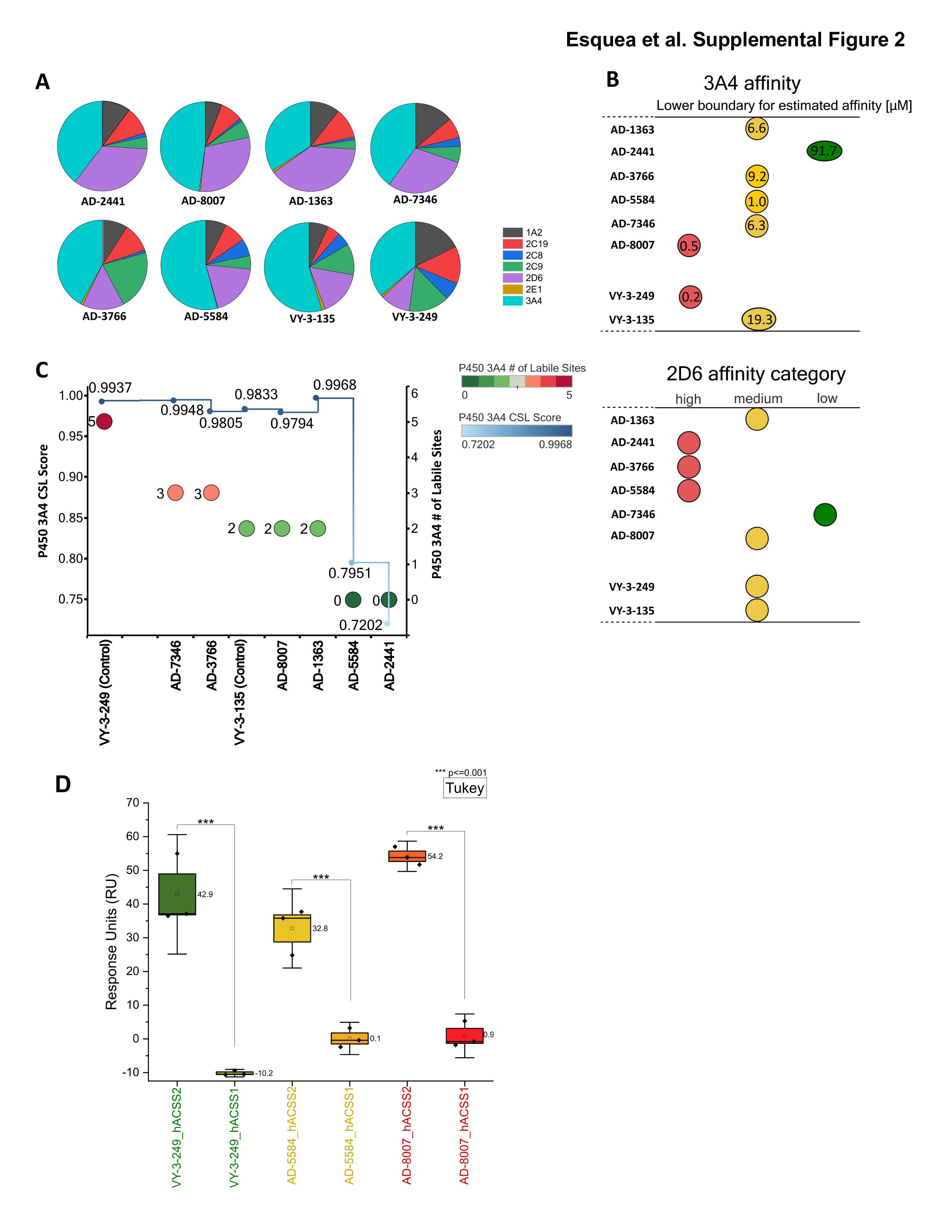

Supplement: Supplementary file 5 [file Image2.JPEG]
