# Supplementary material for: Selective and brain-penetrant ACSS2 inhibitors target breast cancer brain metastatic cells
Source: Front Pharmacol. 2024 May 16;15:1394685. doi: 10.3389/fphar.2024.1394685 (PMC11137182; doi:10.3389/fphar.2024.1394685)
Supplement: Supplementary file 2 [file Table1.PDF]

Supplementary Tables:

Supplementary Table S1: Kinetic and equilibrium parameters for AD-2441 and analogs binding to immobilized ACSS2. Equilibrium Dissociation Constant (K_D) derived from a Langmuir isotherm equilibrium fit or a global fit. Values are mean ± standard deviation (SD) with n=3.

Compound ID	K_D [µM] equilibrium	± SD	k_{on} [$M^{-1}s^{-1}$]	± SD	k_{off} [s^{-1}]	± SD	K_D [µM] kinetic/global	± SD
**VY-3-249 (Control)**	53.2	7.3	1.80E+03	8.93E+02	1.05E-01	2.95E-02	66.0	18.7
**AD-2441**	116.9	21.4	1.46E+03	2.80E+02	1.75E-01	2.89E-02	121.3	14.1
**AD-1363**	130.7	25.9	3.77E+03	1.75E+03	6.34E-01	6.86E-02	208.1	87.7
**AD-8007**	116.6	19.5	4.79E+03	3.10E+03	5.47E-01	2.01E-01	149.4	54.6
**AD-3766**	70.6	24.7	5.01E+03	1.73E+03	3.59E-01	5.55E-02	77.6	20.4
**AD-5584**	188.7	82.8	2.02E+03	6.10E+02	6.32E-01	2.25E-01	352.3	180.3
**AD-7346**	39.8	23.9	1.01E+04	5.17E+03	3.99E-01	1.11E-01	49.4	22.5

Supplementary Table S2: Metabolic Stability of selected ACSS2 inhibitors in human liver microsomes and Plasma stability (WuXi AppTec Co., Ltd.). Testosterone, Diclofenac, Propafenone, and Propantheline Bromide were used as controls.

	Human Liver Microsome Stability						*Human Plasma Stability*
**Compound ID**	**R^2**	**$T_{1/2}$ (min)**	**CLint(mic)(µL/min/mg)**	**CLint(liver)(mL/min/kg)**	**Remaining(T=60min)**	**Remaining(NCF=60min)**	**$T_{1/2}$ (min)**
AD-8007	0.9824	>145	<9.6	<8.6	0.77227	0.95633	>289.1
AD-3766	1	0.914	1516.099	1364.4891	0.0005	0.96386	>289.1
AD-5584	0.983	19.565	70.842	63.7578	0.12368	0.83493	>289.1
Testosterone	0.9884	15.519	89.309	80.3781	0.06959	0.89758	
Diclofenac	0.9939	5.293	261.875	235.6875	0.00044	0.87629	
Propafenone	0.9182	7.919	175.015	157.5135	0.00396	0.93177	
Propantheline Bromide							18.6

Supplementary Table S3: *In vitro* permeability assessment of AD-8007 and AD-5584 in MDR1-MDCK1 cells and appropriate controls (Creative Bioarray, Shirley, USA)

Bi-directional permeability across MDR1-MDCK I cell monolayer - Mimiking the BBB

**Compound ID**	**Mean P_{app} (10^{-6} cm/s)**		**Efflux Ratio**	**Mean %Solution Recovery**		**Note**
	**A to B**	**B to A**		**A to B**	**B to A**	
Nadolol	0.257	ND	ND	99.1	ND	Low permeability marker
Metoprolol	12.9	ND	ND	94.2	ND	High permeability marker
Digoxin	0.0595	7.74	130	84.3	100.4	P-gp substrate
AD-8007	2.81	25.2	8.96	81.3	97.0	AD-8007 has a low efflux ratio and potential to cross the BBB
AD-5584	2.11	44.4	21.0	94.9	111.6	---

ND means not determined
